# An index-based system for early alerts of potential zoonotic disease outbreaks

**DOI:** 10.1371/journal.pone.0356739

**Published:** 2026-08-25

**Authors:** Jaqueline S. Angelo, Livia Abdalla, Douglas A. Augusto, Marcia Chame, Eduardo Krempser

**Affiliations:** Oswaldo Cruz Foundation (Fiocruz), Rio de Janeiro, Brazil; Amity University Amity Institute of Biotechnology, INDIA

## Abstract

The Information System on Wildlife Health (SISS-Geo) is a free platform designed for the real-time collection of georeferenced data on wildlife health and environmental conditions via mobile devices. It serves as a collaborative tool, enabling health professionals, researchers, environmental managers, and the general public to report information about wildlife health occurrences directly within the system. Since its launch in 2014, SISS-Geo has been successfully applied in supporting decision-making during significant wildlife health events in Brazil. In this paper, we introduce a dynamic alert system based on a Multi-Attribute Zoonotic Alert Index (Z-Alert) to enhance disease outbreak detection and response in wildlife. The proposed approach improves the current alert system of the SISS-Geo platform by integrating clustering techniques with multi-objective optimization. The clustering stage groups spatiotemporally related SISS-Geo records, including observations of animals reported as alive, dead, or sick, allowing the identification of patterns relevant for epidemiological surveillance. Each identified cluster is assigned a numerical alert index, the Z-Alert index, reflecting its *level of attention*, based on optimally weighted attributes such as the percentage of dead animals, temporal interval, and geographical spread. The system also offers customization alert thresholds, allowing health managers to adapt the alert system to their specific needs. To validate the model, we used historical Yellow Fever case data from Brazil’s Ministry of Health. By supporting surveillance teams in prioritizing prevention and investigation actions, the system enhances the capacity of public health authorities to respond effectively through supporting risk assessment, optimizing resource allocation, and promoting cost-effective strategies for outbreak control.

## Introduction

It is widely recognized that most emerging zoonotic diseases—those naturally transmissible from animals to humans—originate in wildlife and are driven by human activities like land-use changes, urbanization, and globalization. Emerging infectious diseases (EIDs) are a major global concern. According to [1], approximately 60% of EID events are caused by zoonotic pathogens originating from non-human animal sources, and about 72% of these zoonotic events are attributed to pathogens with a wildlife origin. A recent review highlights the need for enhanced surveillance and multisectoral collaboration for proactively addressing disease outbreaks [2].

Effective surveillance and disease prevention, as well as timely response and control efforts, requires a collaborative, multidisciplinary approach to understand disease ecology and conduct risk assessments. In this context, wildlife surveillance becomes essential for various sectors of society, especially for public health. Collecting and analyzing data on wildlife populations can provide valuable insights to guide preventive actions and control zoonotic disease transmission. The One Health approach is particularly relevant in this context, as it recognizes the interconnection between human, animal, and environmental health [3,4]. This integrated perspective is especially important in a country like Brazil, where environmental and epidemiological complexities require comprehensive strategies for disease surveillance systems [5].

The successful implementation of One Health principles involves multiple dimensions, including political commitment, policy formulation, sustainable financing, knowledge sharing, institutional collaboration, and community engagement [6]. Bringing together various sectors, disciplines, and communities across different levels of society is essential to address both infectious and non-infectious diseases influenced by human activities and environmental change [7]. In addition, we consider that the effective implementation of these principles also depends on the use of innovative technologies that facilitate real-time, high-quality data collection and support decision-making through efficient information analysis and management. In this context, the Brazilian Wildlife Health Information System, SISS-Geo (*Sistema de Informação em Saúde Silvestre Georreferenciado* (in Portuguese) [8,9], was established in 2014 by the Institutional Platform for Biodiversity and Wildlife Health (Pibss) at the Oswaldo Cruz Foundation (Fiocruz) as a practical initiative aligned with these principles, promoting collaborative and participatory monitoring of wildlife health.

Building upon these concepts, SISS-Geo (avaliable at https://www.biodiversidade.ciss.fiocruz.br or https://sissgeo.lncc.br) serves as a digital platform that enables the real-time collection of georeferenced observations on wildlife and environmental conditions via mobile devices, with active public participation. It integrates the registration of occurrences, expert diagnoses, and computational tools for data analysis, prediction, visualization, and real-time alerts for wildlife-related health events. Since its launch, SISS-Geo has proven to be a valuable resource for supporting decision-making during major wildlife health events in Brazil, such as Yellow Fever (YF) outbreaks [10–12], by accelerating information dissemination, providing accurate geographic locations, and simplifying response efforts.

The One Health Joint Plan of Action (2022–2026) document [13] proposed by the Quadripartite Organizations—the Food and Agriculture Organization of the United Nations (FAO), the United Nations Environment Programme (UNEP), the World Organization for Animal Health (WOAH, founded as OIE), and the World Health Organization (WHO)— cites SISS-Geo as an example of an integrated surveillance system that supports early detection and response to zoonotic threats through multisectoral collaboration. This positions SISS-Geo as contributing to the global call for integrated One Health Early Warning and Response Systems (OH-EWRS), particularly by supporting early warning and detection processes through the collection and integration of wildlife health data. Such a role is consistent with existing approaches, such as the Early Warning, Alert, and Response (EWAR), and promotes collaborative surveillance to address emerging and reemerging zoonotic diseases [14,15].

In Brazil, the YF virus is endemic to the Amazon region, eventually reemerging into epidemic waves [11]. While currently confined to South and Central America and parts of Africa, its potential for geographic expansion, raises concerns for public health and cross-border transmission [16]. This underscores the urgent need to develop early warning and response systems capable of detecting outbreaks in their initial stages and enabling timely, coordinated interventions. By enabling the real-time collection of high-quality data, the platform provides critical information to government agencies, the health sector, and society at large—even in some of the most remote regions of the country—thereby strengthening surveillance and response efforts across multiple levels.

One of the most strategic components of SISS-Geo’s features is the alert system, which notifies health managers about reported cases. Currently, the system follows fixed monitoring rules. For instance, if a non-human primate (NHP) death or illness is reported, an email is automatically sent to health managers registered on the platform along with information about the record. While this enables managers to make timely decisions regarding the actions to be taken, it is unable to detect *levels of attention*, as fixed rules fail to account for regional variability and complexity. Without an appropriate prioritization mechanism, it becomes more challenging to direct efforts to the most impactful cases, compromising the efficiency of investigation and intervention measures.

The identification and prioritization of actions in critical health regions are essential for the effective allocation of resources in disease prevention and control efforts. Such an approach prevents the waste of supplies and personnel in low-risk areas, focusing efforts where there is the greatest need. Timely detection of diseases through surveillance, combined with effective implementation of targeted interventions, can significantly reduce the scale, severity, and economic impact of responses to outbreaks [17].

In this study, we aim to leverage data from the SISS-Geo platform to investigate the potential of animal-related information as an early warning signal for disease outbreaks that may later affect humans. As part of the efforts to improve surveillance strategies, we propose an index-based alert system that incorporates regional information to accurately reflect the criticality of events.

The Multi-Attribute Zoonotic Alert Index (named Z-Alert) proposed is a quantitative approach that integrates multiple attributes into a single indicator designed to highlight clusters (group of records in SISS-Geo) potentially associated with elevated health risks. The Z-Alert may guide surveillance priorities by highlighting events of high concern and supporting risk assessment conducted by surveillance teams. By creating the Z-Alert index, we aim to identify not only individual records but also regions that may require increased attention due to potential threats to human and animal health.

This proposal is part of a broader effort to strengthen national surveillance strategies, especially following the expansion of YF into extra-Amazonian regions starting in 2014, which highlighted the need to enhance wildlife health surveillance in Brazil. In this context, tools such as SISS-Geo and the environmental suitability models developed by Pibss, in partnership with the Brazilian Ministry of Health (BMoH) [18], have become central elements for anticipating areas of concern, guiding vaccination efforts, and supporting the definition of ecological transmission corridors. Both have been officially incorporated into national response strategies, including epidemiological bulletins, technical notes [19,20] issued by the General Coordination for Arbovirus Surveillance of the Department of Health Surveillance and Environment of the Ministry of Health (CGARB/SVSA/MS), and the Contingency Plan for Public Health Emergencies – Yellow Fever (2nd edition) [21,22].

Initially developed to support the monitoring of NHPs in the context of endemic YF in Brazil, the SISS-Geo platform has since evolved into a widely adopted tool for wildlife health surveillance, supporting multiple zoonotic diseases nationwide. Its alert system has been widely used for early detection of YF circulation through NHP monitoring. Therefore, as a case study of its effectiveness, we focus on NHP-related events as signals of potential YF circulation, given their key role as sentinels for transmission. By analyzing spatial and temporal patterns of NHPs records, we seek to identify regions that exhibit early signs of disease. Using NHPs as indicators of environmental health risks, encompassing both animal health and the potential transmission to human populations, enables earlier detection and supports timely public health responses before human infections are reported.

Testing the proposed approach in the context of YF as an initial case study, allows us to demonstrate its effectiveness within a well-established nationally relevant surveillance system, validating the potential applicability to other zoonotic diseases. Nevertheless, the proposed methodology is not limited to the YF context, as the SISS-Geo system also contains records related to other infectious diseases, such as rabies and avian influenza. The proposed methodological framework has the potential to be adapted to other zoonotic diseases by adjusting its parameters and reference data to the specific epidemiological and ecological characteristics of each pathogen. However, its applicability to other zoonotic diseases is beyond the scope of the present study and remains to be demonstrated through future research.

Unlike the current rule-based model, the proposed system generates alerts based on clustered data analysis, considering multiple observations recorded in SISS-Geo. By integrating attributes derived from the platform’s georeferenced records, such as the number and proportion of dead animals, event duration, and geographic extent, the system transforms raw groupings of records into structured units that can be meaningfully analyzed.

We combine clustering techniques, to capture spatial and temporal disease occurrence patterns, with an index-based alert system that measures *levels of attention.* A level of attention refers to the ability of the proposed index to distinguish between events that may require varying degrees of priority for surveillance teams. Instead of treating all clusters equally, the proposed alert system ranks clusters based on multiple attributes, allowing the identification of events that may deserve closer monitoring or further investigation.

Such an approach enhances the capacity for early detection and response within the context of zoonotic disease surveillance, representing an evolution of the current rule-based notification scheme of SISS-Geo. The main contribution of the proposed alert system is to support the prioritization of potentially relevant epizootic events, enabling surveillance teams to prioritize investigations, collect samples, and implement timely public health actions. With the aid of a data clustering technique and a multi-objective optimization process, the Z-Alert will integrate multiple attributes to estimate a level of attention associated with a cluster, to support health managers. We also propose customized alert thresholds that dynamically adjust the number of alerts/notifications generated by the system, ensuring that only the most relevant situations are reported to decision-makers.

In this paper, we aim to:

- propose an index-based system that provides a quantitative measure to support risk estimation and prioritization of relevant occurrences. The main idea is to reflect different *levels of attention* in order to offer a more refined assessment of potential health risks. The alert system distinguishes these levels of attention by incorporating multiple regional attributes.
- design the alert system in a flexible way that can be later customized by health managers to prioritize alerts and optimize investigation and intervention efforts according to their specific needs and realities. In a vast country like Brazil, where environmental, epidemiological, and logistical conditions vary widely, such flexibility is essential for efficient resource allocation.
- apply a clustering method to capture spatial and temporal patterns of disease occurrence, enabling a more comprehensive analysis of outbreak dynamics.
- design a multi-objective optimization process to determine the optimal combination of attribute weights in the alert index, balancing key criterias to ensure accurate alerts that minimize false negatives to prevent the non-identification of relevant clusters while reducing false positives to avoid overloading verification systems.
- provide a comprehensive analysis of the results obtained from the multi-objective optimization process and validation of the Z-Alert Index using data from the Brazilian Ministry of Health (BMoH), which comprises records on confirmed human cases and NHP epizootics of YF in the national territory.

The effectiveness of the proposed system relies heavily on the accuracy of its prediction model in correctly identifying both true positives (alerts with a confirmed case of a disease) and presumed true negatives (non-alerts). Missing an alert condition (false negative) can have serious consequences for wildlife, the environment, and human health. Conversely, false positives could overwhelm the limited network of laboratories and experts responsible for verifying alerts. However, this effect can be controlled by adjusting the sensitivity of the Z-Alert index, supporting the prioritization process by helping surveillance teams filter and rank alerts before further investigation. Thus, finding the right balance between classifying alerts and non-alerts is crucial to ensuring that the system effectively prioritizes critical regions without overloading resources. In addition, the customization of alert thresholds plays a key role in this trade-off, as stricter limits reduce false positives but increase the risk of missing real cases (less sensitivity), while more relaxed limits enhance detection at the cost of more false alerts (more sensitivity).

Ultimately, the outcome of this work will also contribute to the integration of human, animal, and environmental health data, supporting the One Health approach to improve zoonotic disease surveillance and prevention.

### Proposed methodology

The concept of the Z-Alert Index is to provide early warnings based on the level of attention associated with a group of records in SISS-Geo. This level of attention can be interpreted as a form of triage, as the system is designed to issue alerts for clusters that reach a specific alert threshold, which can be customized by health managers. The proposed system aims to support validation by human experts and prioritization of relevant events. Each alert will still be verified by health managers before any action is taken, ensuring that decisions remain under expert assessment and validation.

The proposed methodology integrates data preprocessing and organization of SISS-Geo records, including filtering and attribute extraction, a clustering technique, and a multi-objective optimization approach. The index-based alert system utilizes regional information to quantify the level of attention associated with an *event*. An event is any occurrence or signal that may indicate a potential risk to public health and requires further investigation. In this context, a group of records (cluster), representing wildlife observations (including animals reported as alive, dead, or sick), is a sentinel for events, which in turn is based on occurrence patterns in both space and time.

By clustering such occurrences and quantifying their attributes through the alert index, it becomes possible to prioritize areas where the risk is escalating, even when individual reports may seem sparse or dispersed. In this sense, the index can highlight clusters of potential concern without relying on predefined criteria, thereby complementing the current SISS-Geo’s alert system.

**Fig 1:**
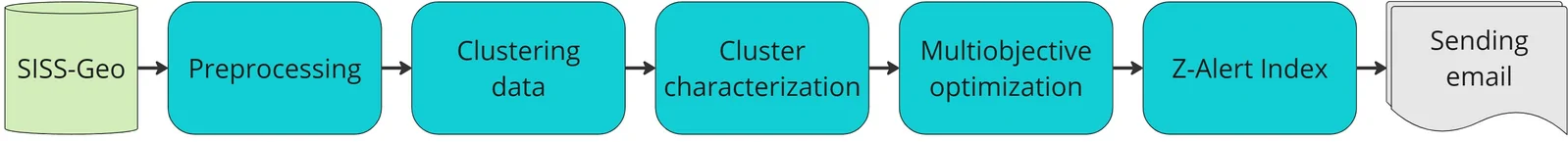
The zoonotic alert system. Main steps of the zoonotic alert system.

The system relies on the following main steps, as illustrated in Fig 1. Initially, data obtained from SISS-Geo are filtered according to the event of interest, where typically only a subset of host animal types is relevant for the analysis (Section Data preprocessing). Then, a clustering technique is applied to group spatiotemporally related records into clusters (Section Clustering data). After clustering, new attributes are extracted from the records to characterize each cluster in greater detail (Section Cluster characterization). In the optimization step (Section Multiobjective optimization problem (MOP)), a multi-objective optimization process is defined based on these cluster attributes, with the goal of obtaining an alert index capable of differentiating between *critical* and *non-critical* clusters (as defined in Section Optimizing cluster attribute weights). Next, the optimization process assigns weights to each attribute, determining its relevance in calculating the Z-Alert index. Finally, the Z-Alert index is assigned to each cluster (Section The Z-Alert Index), reflecting its level of attention. Health managers can then define a threshold level (defined in Section Threshold Level) for receiving alerts (e.g., via email notification), which can be adjusted according to operational needs. Clusters exceeding this threshold are highlighted, and automatic notifications are sent to managers to prioritize alerts and support timely investigation.

### Data preprocessing

The first step is the filtering of SISS-Geo’s data according to the event of interest. The data collected are described in Table 1, which summarizes all filters applied to obtain the dataset from the SISS-Geo platform. The clustering process was based on spatiotemporal attributes (geographic coordinates and date), while other variables, such as animal condition and disease classification, were used in subsequent stages for cluster characterization.

**Table 1:** Data collected from the SISS-Geo platform.

Field	Descriptor field
Record	Identifier, Longitude, Latitude, Observation date (ISO), State, Municipality, Source of location and Precision.
Record status	“Checking with experts” (records verified by the SISS-Geo internal team and awaiting validation by a taxonomist), “Audited” (records verified by the SISS-Geo internal team), and “Valid” (records validated and approved by taxonomist).
Animal	Identifier, Type, Observed quantity, Animal condition (alive or dead), and Behavior (if alive, normal or sick).
Final classification	Disease (disease under investigation) and Classification (positive or negative for the tested disease).

In the preprocessing stage, only records obtained via GPS or with locations explicitly provided by the user were selected, on top of having locations errors within 100 m to ensure the spatial reliability of the data used for cluster formation. After data collection and filtering, each record was geocoded to its corresponding municipality using latitude and longitude coordinates, based on the official dataset provided by the Brazilian Institute of Geography and Statistics (IBGE) (available at https://geoftp.ibge.gov.br/organizacao_do_territorio/malhas_territoriais/malhas_municipais/municipio_2022/Brasil/BR/BR_Municipios_2022.zip). This information is necessary for validating the results presented in section Validation of the Z-Alert Index.

It is important to note that, although the publicly available SISS-Geo interface provides access to a limited subset of the data (e.g., location, date, and species), the full dataset used in this study includes additional information, such as disease outcomes, and confirmed cases, accessible only to authorized institutional users due to legal and privacy constraints, according to the Data Availability statement.

### Clustering data

Clustering techniques are designed to identify instances or groups of data that belong to the same phenomenon. The idea is to separate data with similar characteristics and assign them to a group or cluster. Given the dynamic nature of the phenomenon of interest, such as the occurrence of disease outbreaks, these events exhibit spatial and temporal movement patterns [12]. In this context, clustering enables the identification of groups of records that share these dynamic characteristics, supporting a more refined analysis of disease spread.

In this study, the DBSCAN [23,24] was selected due to its ability to identify clusters of arbitrary shapes, its robustness to noise and outliers, and its minimal parameter requirements. Additionally, unlike many clustering methods, DBSCAN does not require prior specification of the number of clusters, which is particularly advantageous in exploratory analyses where the underlying structure is unknown.

### Spatiotemporal Distance matrix

The distance matrix assigned to DBSCAN was generated by combining two matrices: (i) a spatial distance matrix $D_{S}$, which considers the geodesic distance between points. The geodesic distance is calculated in a three-dimensional spherical space as the shortest path along the curved surface of the planet. This distance is computed based on the latitude and longitude of the points, and (ii) a temporal distance matrix $D_{T}$, which indicates the time in days between records. Note that both $D_{S}$ and $D_{T}$ are square matrices, which is a prerequisite of the *metric* parameter.

The spatial and temporal constraints are considered when computing both matrices, with the temporal and spatial limits configured according to the experiments conducted (see section Experimental Results). The matrices $D_{S}$ and $D_{T}$ are computed as follows. Let $D_{S}(i,j)$ be the geodesic distance between points *i* and *j*. If $D_{S}(i,j)\geq L_{D_{S}}$, where $L_{D_{S}}$ is the maximum spatial limit allowed for clustering points, a high value is assigned to indicate that these points cannot be grouped. Otherwise, $D_{S}(i,j)$ is assigned the actual distance between *i* and *j*. Similarly, let $D_{T}(i,j)$ be the temporal distance between instances *i* and *j*. If $D_{T}(i,j)\geq L_{D_{T}}$, where $L_{D_{T}}$ is the maximum temporal limit allowed for clustering, a high value is assigned to indicate that these instances cannot be grouped. Otherwise, $D_{T}(i,j)$ is assigned the time interval (in days) between *i* and *j*.

Since $D_{S}$ and $D_{T}$ have different scales (space versus time), both were normalized (values were scaled in the interval [0,1]) to prevent large values from distorting the clustering process. The limits $L_{D_{T}}$ and $L_{D_{S}}$ are defined by the user according to the experiments performed. Finally, the total distance matrix $D_{total}$ used as input for DBSCAN is computed as


$$\begin{array}{c} D_{total}=D_{S}+D_{T} \end{array}$$

As a result of the clustering process, we obtain the clusters to which each record belongs, respecting the predefined temporal and spatial limits. It is important to highlight that the temporal and spatial limits are established for pairs of points, meaning that the generated cluster is not necessarily restricted to these limits. In other words, this implies that a cluster may extend beyond the initial limits if additional records are connected through intermediate links. For instance, if record A is within 1 km and 30 days of record B, and record B is within the same limits of record C, all three records will belong to the same cluster, even if A and C are more distant from each other. This chaining effect enables DBSCAN to capture the continuity of spatiotemporal patterns, which may reflect the spread or persistence of health-related events.

### Cluster characterization

After the clustering step, new information is assigned to each cluster to support the construction of the Z-Alert index. The newly extracted attributes are presented in Table 2.

**Table 2:** Extracted attributes used to characterize clusters.

Attribute	Description
n_death	Number of dead animals.
n_animal	Total number of animals involved; sum of live, sick and dead animals.
ini_date	Cluster start date, corresponding to the oldest record.
end_date	Cluster end date, corresponding to the most recent record.
*interval*	Time interval (in days); difference between the dates of the earliest and most recent records.
n_rec	Number of records.
freq_rec	Frequency of records; number of records divided by *interval*.
freq_death	Frequency of dead animals; number of dead animals divided by *interval*.
freq_animal	Frequency of total animals; number of animals involved divided by *interval*.
perc_death	Percentage of dead animals; number of dead animals divided by n_animal.
*extention*	Maximum distance between all points in the cluster.
n_conf	Number of animals confirmed to have a disease of interest.
*geocode*	Cluster geocoding.

The cluster geocode is determined based on the geocodes of the records it comprises. If a cluster contains records from different municipalities (i.e., with distinct geocodes), it is duplicated in the database to ensure that each entry maintains the correct association with its respective municipality. This information is only necessary to validate the results (see in section Validation of the Z-Alert Index).

### Multiobjective optimization problem (MOP)

Multi-objective optimization (MOP) aims to simultaneously optimize multiple objectives, potentially conflicting, generating a set of trade-off solutions that support decision-making in complex scenarios. Such a set of solutions, also called Pareto front or non-dominated solutions, represents the best collection of solutions found for the problem. For further details on MOP, readers are referred to [25].

### Optimizing cluster attribute weights

The goal of the Z-Alert index is to generate meaningful insights from data groups rather than individual records, enabling an accurate determination of a cluster’s criticality in relation to potential phenomena of interest. Thus, in the optimization phase, only clusters with more than one record were considered, and among these, only those containing confirmed cases of a disease of interest were selected. There are three primary reasons for considering only multi-record clusters: (1) in SISS-Geo, there is already a rule-based alert system triggered by individual records; (2) in geometric terms, determining the event spatial direction requires at least two records; and (3) cluster characterization is only statistically meaningful when more than one sample is available. Clusters with confirmed cases were classified as *critical clusters*, while clusters without confirmed disease cases were classified as *non-critical clusters*. The formulated MOP aims to optimize two objectives simultaneously:

(i) The mean of the critical clusters, which needs to be maximized, and
(ii) The variance of the critical clusters, which needs to be minimized.

By using a weighting method [25], the aim is to maximize a function that represents the combination of these two objectives. This ensures that the alert index effectively distinguishes critical clusters from non-critical ones while maintaining uniformity among the critical clusters. The objective function that we aim to maximize for optimizing the clusters’ attribute weights is


$$\begin{array}{c} F=w\cdot f_{1}\left(Z\_Alert,n\_death\right)-(1-w)\cdot f_{2}\left(Z\_Alert\right) \end{array}$$ (1)

where Z_Alert (defined in the next section) are the indices of critical clusters, $f_{1}\left(Z\_Alert,n\_death\right)$ represents the mean of the indices of critical clusters, weighted by the number of dead animals (n_death), and $f_{2}\left(Z\_Alert\right)$ represents the variance of the indices of critical clusters. The parameter w≥0 controls the importance of maximizing *f*_1_, while (1−w) controls the importance of minimizing *f*_2_, ensuring that w+(1−w)=1. Objectives *f*_1_ and *f*_2_ are computed as


$$\begin{array}{c} f_{1}(Z\_Alert,n\_death)=\frac{\sum_{i=1}^{nc}Z\_Alert[i]\cdot n\_death[i]}{\sum_{i=1}^{nc}n\_death[i]} \end{array}$$ (2)

$$\begin{array}{c} f_{2}(Z\_Alert)=\frac{1}{nc}\sum_{i=1}^{nc}(Z\_Alert[i]-\mu {)}^{2} \end{array}$$ (3)

where *nc* is the total number of critical clusters and μ is the mean of Z_Alert of critical clusters. By maximizing the function (1), we encourage the attribute weights to adjust the index according to both combined objectives.

### The Z-Alert index

For the construction of the alert index, let *M* be the attribute matrix of the clusters, composed of the columns


$$\begin{array}{c} M=[n\_rec,interval,freq\_n\_rec,freq\_a\_animal,extention,perc\_death,n\_death] \end{array}$$

and let $p\in {\mathbb{R}}^{m}$, where *m* is the number of variables, be the corresponding weight vector, which characterizes the importance of each attribute in computing the index. Due to the differences in scale among the variables, a data standardization process was applied to ensure that all variables contribute equally to the optimization step. The adopted standardization is based on the *Z-score* normalization, which transforms the data into a distribution with a mean zero and a standard deviation of one, calculated as


$$\begin{array}{c} Z=\frac{X-\mu }{\sigma } \end{array}$$ (4)

where *Z* is the normalized value, *X* is the original value of the variable, and μ and σ are the mean and the standard deviation of the variable, respectively.

Finally, for each cluster *i* = 1,...,*n*, where *n* is the total number of clusters, the Z_Alert index is calculated as


$$\begin{array}{c} Z\_Alert[i]=\sum_{j=1}^{m}M[i,j]\cdot p[j] \end{array}$$ (5)

where *p*[*j*] is the weight associated to variable *j*, and $M\in {\mathbb{R}}^{n\times m}$ is the matrix in which column *j* represents the value of the *j*-th variable and line *i* represents the *i*-th cluster.

At this stage, each cluster will receive a Z-Alert index, reflecting its level of attention. Clusters with low index values are considered lower risk, while those with high index values indicate greater risk. However, the interpretation of this level of criticality may vary depending on the reality of each region, under the management of health departments. To accommodate this flexibility, the health manager can define a threshold level for receiving alerts, called threshold_level, which can be adjusted according to their needs and preferences.

### Threshold level

While the Z-Alert assigns a numerical value to clusters based on their characteristics, the threshold level is a configurable parameter used to determine which clusters generate alerts. It establishes a threshold for cluster criticality, directly influencing the number of alerts/notifications generated and ensuring that only the most relevant situations are reported to the manager. Considering that mortality records may be subject to under-reporting, the alert index is designed to mitigate this by the aggregation of multiple attributes at the cluster level, to provide a more robust signal. By calibrating the threshold appropriately, the system reduces the likelihood of overreacting to low-risk events while still capturing emerging patterns that may not be apparent from individual reports alone.

To define this threshold in an objective and adaptable manner, we use the distribution of the Z-Alert indices and perform the cutoff based on quartiles. Quartiles are statistical measures that divide an ordered set into four equal parts, each containing 25% of the observations. In the proposed scheme, quartiles are flexible, allowing the health manager to set the most appropriate threshold level for their reality. For instance, by choosing threshold_level=0.9 as the limit, only the top 10% most critical clusters will generate alerts, ensuring greater focus on high-risk regions. If a broader criterion is needed, the manager can opt for a lower quartile, increasing the number of received notifications. This approach provides greater flexibility and customization, allowing the system to adapt to different regional demands. In practice, alert thresholds are empirically defined by health managers through an iterative, trial-and-error process. Since this threshold directly affects the number of generated alerts, the appropriate value is determined according to the specific needs and operational realities of each surveillance context.

In the next section, the computational experiments will be presented, where different performance metrics were used to analyze the quality of the obtained solutions, evaluating the effectiveness of the Z-Alert index in distinguishing between critical and non-critical clusters and its impact on prioritizing notifications.

### Experimental results

As a case study, we utilize data from NHPs in SISS-Geo platform for identifying clusters potentially associated with YF virus circulation. It is important to highlight that, although the national surveillance system recommends investigating a single dead or illness NHP, the alert index does not replace this approach—it complements it. While isolated cases are critical for early detection, clusters of NHP deaths often indicate that transmission is already underway.

In this section, we validate the results obtained by the Z-Alert index, by comparing them with official YF records from the Brazilian Ministry of Health (BMoH) [26]. The BMoH dataset, covering the period from 05 May 2014–31 December 2024, was used exclusively for validation purposes. This initial date coincides with the first valid record from NHPs in SISS-Geo, while the final date reflects the ministry’s latest update on YF case occurrences in Brazil. The database provided by the BMoH contains individualized and anonymized data on confirmed human cases and NHP epizootics of YF, with dates of symptom onset or occurrence and with the probable place of infection located within the national territory.

The following criteria were considered for data collection:

Animal type: Marmoset and monkey.

Inclusion period: from 05 May 2014–31 December 2024.

Collection date: 04 April 2025.

As a result of this search, we obtained 6831 records. These data were filtered, considering only records obtained via GPS or explicitly provided by the user, with an accuracy of 100 m to filter out imprecise geolocations, resulting in 4821 records for analysis. After data collection and filtering, each record was assigned the geocoding of the municipalities based on latitude and longitude information. This geolocation step was necessary only for validating the results, as the data provided by the BMoH reports YF cases at the municipal level.

Next, the clustering phase was performance, where the parameters used in DBSCAN were set as follows:

*eps = 500,* which defines the neighborhood radius, controlling the spatial and temporal extent of clusters based on the precomputed distance matrix*.*

*min_samples = 1,* that allows the inclusion of isolated records, reflecting the nature of SISS-Geo data, where events may occur sparsely in space.

*metric = ‘precomputed’,* indicating that clustering is performed using a precomputed distance matrix that integrates spatial and temporal information.

**Fig 2:**
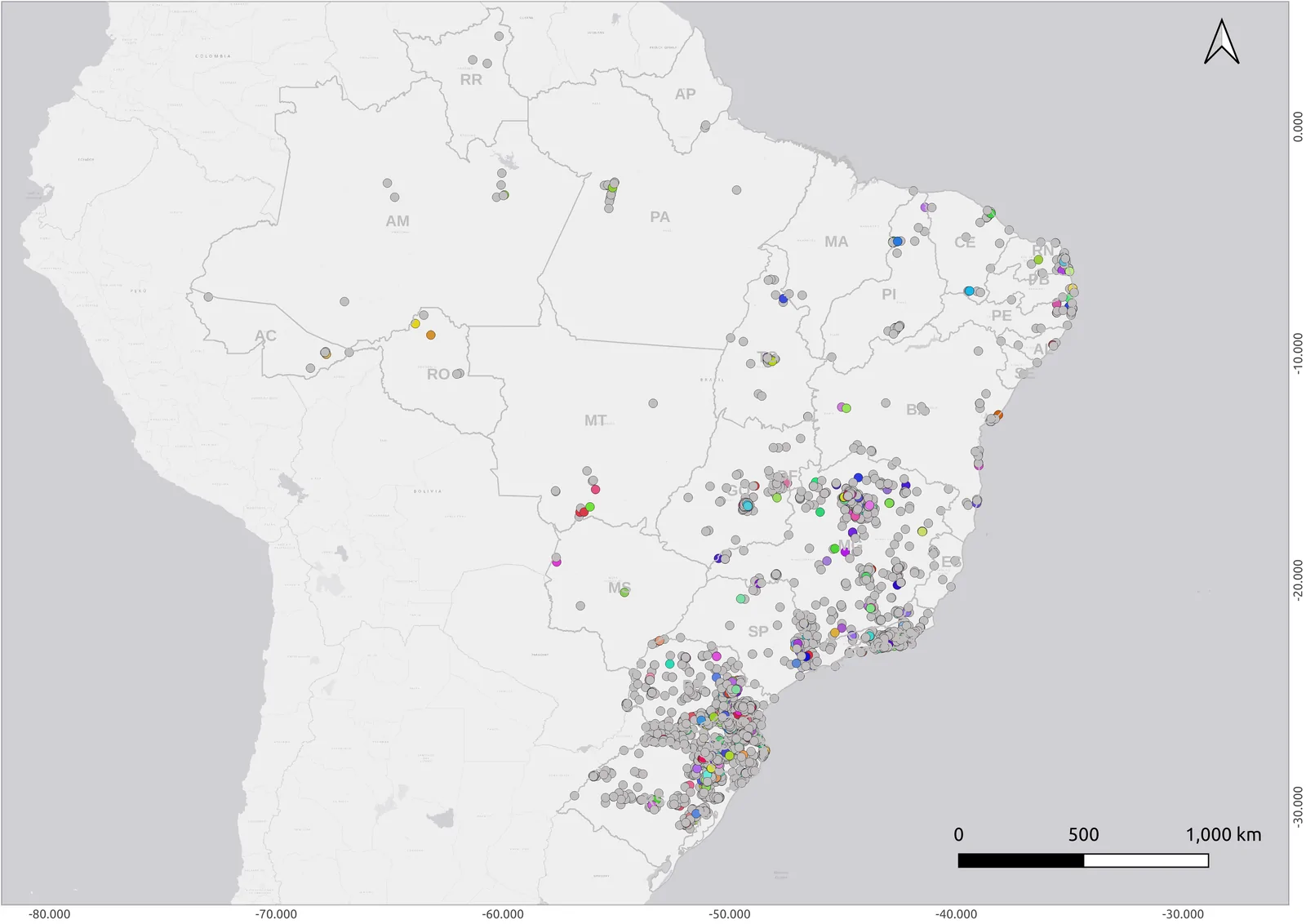
Data clustering. Illustration of all clusters obtained, where unit clusters are colored in gray circles. *Map image is the intellectual property of Esri and is used herein under license. Copyright © 2025 Esri and its licensors. All rights reserved. Source: Esri (ArcGIS Online). Tile service: https://services.arcgisonline.com/ArcGIS/rest/services/Canvas/World_Light_Gray_Base/MapServer. Terms of use: https://doc.arcgis.com/en/arcgis-online/reference/static-maps.htm*.

**Fig 3:**
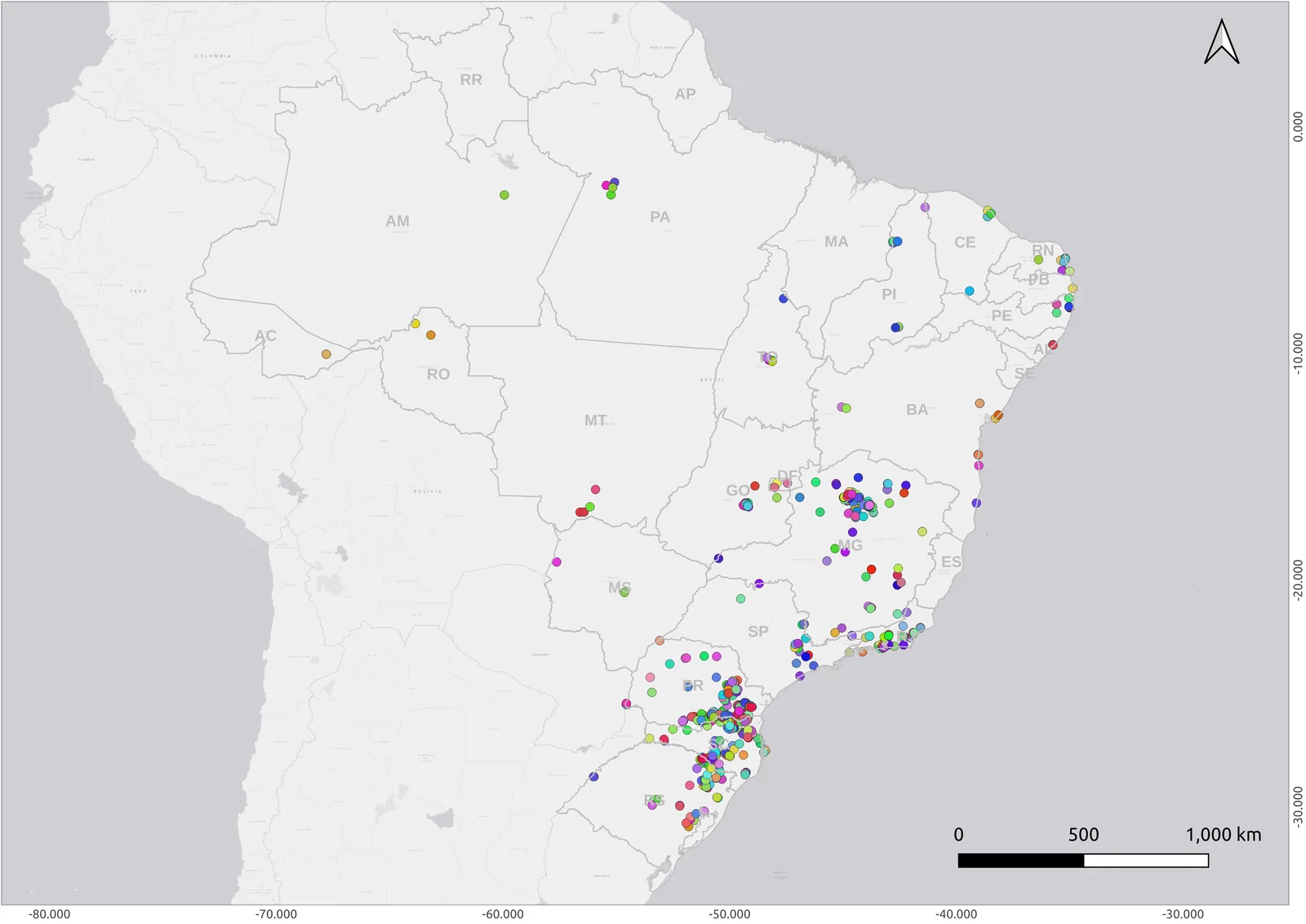
Data clustering. Emphasizing only non-unit clusters. *Map image is the intellectual property of Esri and is used herein under license. Copyright © 2025 Esri and its licensors. All rights reserved. Source: Esri (ArcGIS Online). Tile service: https://services.arcgisonline.com/ArcGIS/rest/services/Canvas/World_Light_Gray_Base/MapServer. Terms of use: https://doc.arcgis.com/en/arcgis-online/reference/static-maps.htm*.

Figs 2 and 3 present the clusters generated after the clustering step, using the limits $L_{D_{S}}=1$ km and $L_{D_{T}}=30$ days. The clustering process resulted in 3508 clusters, of which 2841 were unit clusters, representing approximately 81% of the total clusters. Clustering parameters require careful setting, as they directly influence cluster formation and, consequently, the resulting alert index values.

In this study, the spatial and temporal constraints were empirically defined, as it was difficult to establish a strictly biological or conceptual definition for the parameters, given the characteristics of the available data. These include potential uncertainties in records and imbalance in the spatial distribution of records across regions, where records are more densely concentrated in the southern and southeastern regions of Brazil, as illustrated in Fig 2. Therefore, different combinations of parameters $L_{D_{S}}$ (500 m, 1 km, 3 km) and $L_{D_{T}}$ (10 days, 30 days) were explored to assess their impact on cluster formation. We observed that reducing these limits leads to an increased number of unit clusters and more fragmented groupings, as fewer records satisfy the minimum density criteria required by the clustering algorithm. Conversely, larger limits tend to merge distinct events into broader clusters. Hence, the selected values represent a trade-off between preserving meaningful spatiotemporal aggregation and avoiding excessive fragmentation of clusters. In S1 Appendix, we provide a sensitivity analysis of the clustering parameters $L_{D_{S}}$ (500 m, 1 km, 3 km) and $L_{D_{T}}$ (10 days, 30 days), evaluating six parameter combinations and comparing their performance with the reference configuration (30 days, 1 km), to support the chosen parameter values. S1 Fig compares the Pareto fronts obtained for the six combinations of spatial and temporal clustering parameters and S2 Fig summarizes the relative differences in performance with respect to the reference configuration.

In the next stage, cluster characterization was performed, where new attributes were generated, as presented in section Cluster characterization. At this stage, relevant information is assigned to each cluster to support the construction of the Z-Alert index and to prepare the cluster attributes for the subsequent optimization phase.

### Analyzing the Pareto Front

As described in section Optimizing cluster attribute weights, only clusters with more than one register were considered for the optimization process, and among these, only those containing confirmed YF records were included. This is particularly relevant for NHPs records, since most of the generated clusters, approximately 81%, contain only a single record (*unit clusters*). If all these clusters were included, the Z-Alert index would be biased toward unit clusters, which provide little regional information about the analyzed attributes compared to clusters with multiple records. Thus, for the attribute weights optimization process, 264 records grouped into 38 clusters were considered. To maximize the objective function *F* described in equation (1), the SLSQP (Sequential Least Squares Programming) method implemented in the SciPy library (https://scipy.org/) was used. The bounds on the variables are $0\leq p_{j}\leq 1$ for all j=1,...,m, constrained by $\sum_{j=1}^{m}p_{j}=1$. Different values of *w* were tested for generating the Pareto front. The parameter *w* controls the priority between the two optimization objectives:

- Higher values of *w* assign greater weight to the mean of critical clusters indices, emphasizing the maximization of alerts in high-risk regions.
- Lower values of *w* assign more importance to the variance of critical cluster indices, aiming to reduce value dispersion and ensure a more balanced distribution of alerts.

The tested values were


$$\begin{array}{c} w=[0,0.1,0.2,0.3,0.4,0.5,0.6,0.7,0.8,0.9,1] \end{array}$$

**Fig 4:**
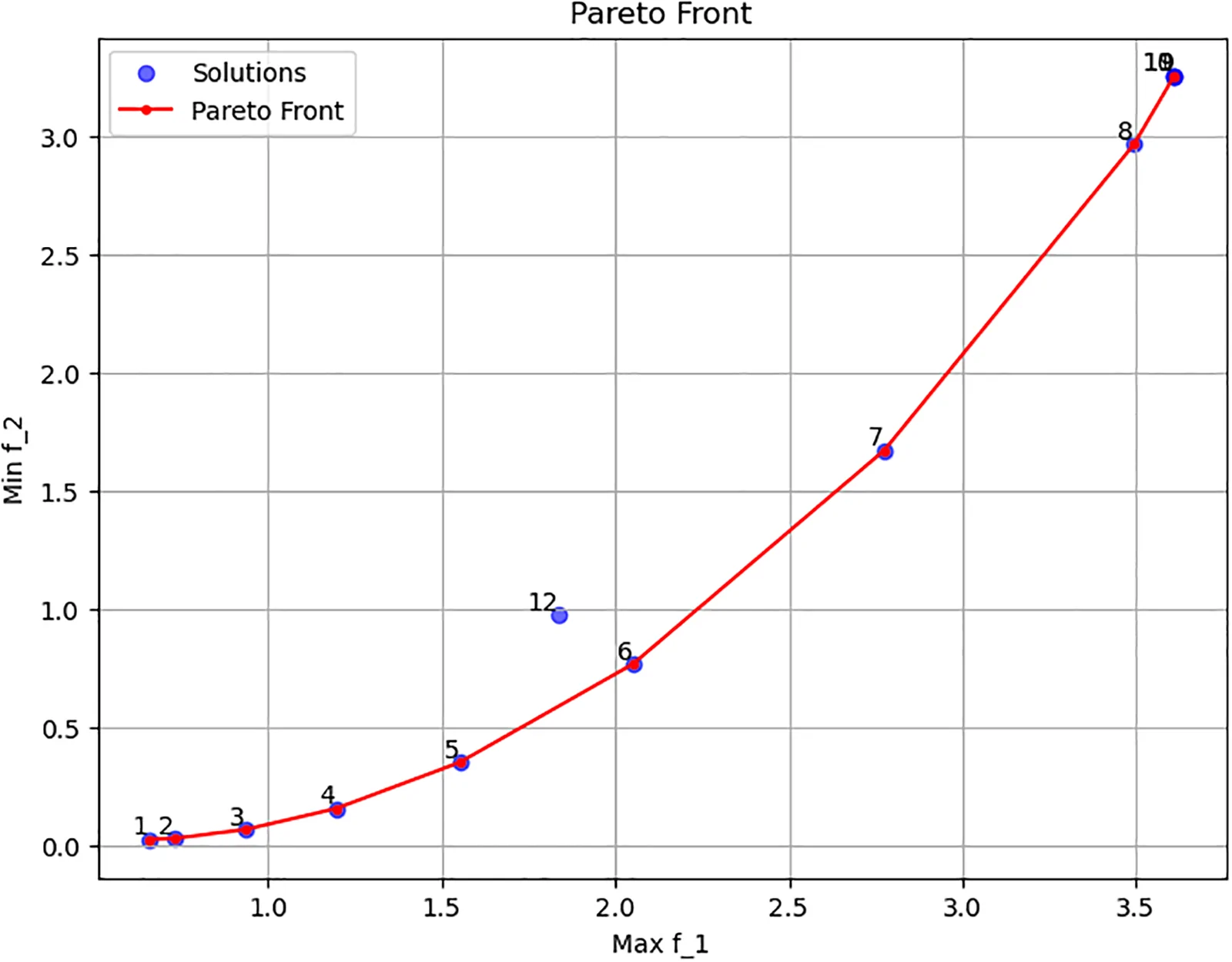
Pareto front. Pareto front obtained.

Additionally, we evaluated a scenario in which no optimization process was used for weight assignment. In this case, the weights were evenly distributed among all variables, assuming that each attribute has the same importance in constructing the Z-Alert Index. The SLSQP method was executed 11 times, once for each value of *w*, with *w* = 0 for the first experiment, *w* = 0.1 for the second, and so on. The non-optimized experiment was also considered, totaling 12 experiments. Each execution resulted in different values for the analyzed objectives. Fig 4 presents the obtained Pareto front, where objective *f*_1_ is maximized and *f*_2_ is minimized. The values from 1 to 12 indicate the solutions obtained, representing the experiments conducted.

From these results, we can conclude the following points:

- The resulting front exhibits the expected behavior for a multiobjective optimization problem, where there is a trade-off between the two objectives [25]. As *f*_1_ improves (increases), *f*_2_ worsens (also increases). The red-connected points represent the Pareto front, meaning they are non-dominated solutions where no objective can be improved without compromising another. Note that uniformly distributing the values of *w* does not necessarily result in a uniformly distributed set of solutions on the Pareto front, as observed in the figure.
- The solution marked as 12 does not belong to the Pareto front, indicating that it is dominated by another solution. This means that assigning uniform weights to the attributes does not produce an efficient solution, reinforcing the importance of optimization in determining the weights.
- Solutions 9, 10, and 11, which correspond to *w* = 0.8, 0.9, 1, produced the same results. This suggests that the maximization of *f*_1_ reaches a limit, where further variations in *w* no longer affect model performance.
- The best solution depends on the application’s priority. If the goal is to only maximize *f*_1_, a point further to the right on the curve should be selected. If minimizing *f*_2_ is more important, the leftmost point is preferable. If the objective is to maintain a balanced index for critical clusters without excessively increasing variance, the optimal point should be near the middle of the curve, suggesting that experiment 6 is the best alternative, as it keeps *f*_1_ reasonably high while significantly improving *f*_2_.

### Analyzing the attributes’ weights

The attribute weights obtained in each solution are presented in Table 3. This table allows for a more detailed analysis of the relationship between attribute weights and objective values. From these results, we can highlight the following points:

- Solution 12 (highlighted in the table) that applies uniform weights without optimization, presents a uniform distribution of weight values leading to a dominated solution in the objective space. This outcome highlights the importance of weight optimization, as it allows the alert index to better differentiate between clusters by emphasizing attributes that are more strongly associated with critical clusters. By assigning weights based on their contribution to relevant patterns, the optimized solution achieves improved performance in identifying high-risk areas.
- Solutions 9, 10, and 11 prioritize only the n_death attribute, assigning 100% of the weight to it. These experiments achieve the best value for *f*_2_, indicating that n_death is the most relevant attribute. However, since objective *f*_1_ worsens significantly, these solutions are not suitable if a trade-off must be maintained.
- Solution 7 and 8 prioritize the n_death attribute but also introduce weights for freq_rec and *extention*. The perc_death attribute also shows relevance in experiment 7.
- Solutions 5 and 6 generate good trade-off solutions between *f*_1_ and *f*_2_. Both indicate that perc_deaths plays a crucial role in balancing the objectives, with n_death being the second most important attribute. Other attributes assigned weights in these experiments include n_rec, *interval*, freq_rec, and *extention*.
- Solutions 1–4 prioritize the minimization of *f*_2_, where perc_death emerges as the most relevant attribute, followed by *interval*, freq_animal, and n_rec.

**Table 3:** Assignment of attribute weights and objective values in each experiment.

Sol.	n_rec	*interval*	freq_rec	freq_animal	*extention*	perc_death	n_death	*f*_1_	*f*_2_
1	0.007	0.140	0.000	0.097	0.000	0.756	0.000	0.655	0.027
2	0.041	0.127	0.002	0.061	0.000	0.768	0.000	0.732	0.032
3	0.047	0.120	0.016	0.000	0.000	0.761	0.056	0.935	0.069
4	0.048	0.101	0.018	0.000	0.005	0.688	0.141	1.195	0.157
5	0.040	0.067	0.024	0.000	0.032	0.581	0.256	1.551	0.355
6	0.028	0.020	0.033	0.000	0.070	0.432	0.416	2.050	0.769
7	0.000	0.000	0.055	0.000	0.109	0.185	0.651	2.775	1.673
8	0.000	0.000	0.034	0.000	0.039	0.000	0.927	3.495	2.968
9	0.000	0.000	0.000	0.000	0.000	0.000	1.000	3.611	3.257
10	0.000	0.000	0.000	0.000	0.000	0.000	1.000	3.611	3.257
11	0.000	0.000	0.000	0.000	0.000	0.000	1.000	3.611	3.257
12	0.143	0.143	0.143	0.143	0.143	0.143	0.143	1.839	0.977

Observe that the attributes n_death (number of dead animals) and perc_death (percentage of deaths) capture complementary aspects of disease severity. While the absolute count provides a direct measure of mortality events, the latter highlights the lethality in proportion to the affected population, thereby enabling a more robust characterization of epidemiological risk. The presence of perc_death seems to be crucial for determining the Z-Alert Index, as it appears to influence both objectives in a more balanced and controlled manner than n_death. This outcome is expected, given the structure of the objective functions, which are formulated based on critical clusters. In these clusters, animals are confirmed to be infected with the YF virus and are therefore likely deceased. The other attributes show a certain degree of importance, appearing in a balanced way in most of the experiments.

### Validation of the Z-Alert Index

In this section, the Z-Alert Index is validated using data from the BMoH containing records of YF cases within a predetermined period. The goal is to verify whether the alerts generated by the index correspond to the cases recorded in the BMoH database. Validation will be carried out through the geocoding of municipalities and the analysis of the event occurrence period. To confirm an alert, we consider a 30-day window validation. Specifically, when an alert is generated at time *t*, we search for confirmed cases within a forward validation window (e.g., from *t* to *t* + 30 days). Consequently, the reported performance reflects the ability of the system to identify events before official case confirmation, rather than after confirmation had already occurred. The 30-day window reflects the typical delay period between field detection, laboratory diagnosis, and the final confirmation of a case at the municipal, state, and federal levels. This period provides a realistic buffer to account for reporting and confirmation delays.

To assess the performance of the Z-Alert Index, we use a confusion matrix [27]. The confusion matrix is a widely used tool to visualize the performance of classification algorithms. It organizes the results in a table that details the relationship between the model’s predictions and the actual observed values, as shown in Table 4.

**Table 4:** Confusion matrix.

	Predicted Positive	Predicted Negative
**Actual Positive**	True Positive (TP)	False Negative (FN)
**Actual Negative**	False Positive (FP)	True Negative (TN)

In this study, the class defined as *actual* represents the data from the BMoH database containing confirmed YF cases. A municipality is classified as positive if a confirmed case of the disease is present, whether in humans or non-human primates. Thus, all records in the BMoH database correspond to positive cases. The class defined as *predicted* represents the alerts generated by the system. A cluster is classified as positive if it triggers an alert and negative otherwise. Based on this, the classification categories are defined as follows

- True Positive (TP): The system generates an alert, and the corresponding municipality has a confirmed YF case within the same period.
- False Negative (FN): The system does not generate an alert, but the corresponding municipality has a confirmed YF case within the same period.
- False Positive (FP): The system generates an alert, but the corresponding municipality does not have any confirmed YF cases within the same period. Notice that this is not a quite reliable metric due to probable under-reporting; in other words, the metric tends to be pessimistic.
- True Negative (TN): The system does not generate an alert, and the corresponding municipality also does not have any confirmed YF cases within the same period. Analogously, this metric may be impacted by under-reporting.

In the context of surveillance data, the identification of true negatives is inherently challenging due to reporting delays and potential underreporting. Consequently, the evaluation of non-alerts, actually presumed true negatives, should be interpreted with caution, as the absence of confirmed cases does not necessarily imply the absence of disease.

Based on this classification, various performance metrics were employed to evaluate the proposed alert system. The metrics used are described below:

- Precision: measures the accuracy of positive predictions, defined as: $Prec=\frac{TP}{\left(TP+FP\right)}$
- Sensitivity: is the true positive rate, i.e., the proportion of correctly classified positive predictions, calculated as: $Sen=\frac{TP}{\left(TP+FN\right)}$
- F1-Score: corresponds to the harmonic mean between Precision and Sensitivity, expressed as: $F1=\frac{2(Pr\cdot Sen)}{\left(Pr+Sen\right)}$
- Specificity: is the true negative rate, i.e., the proportion of correctly classified negative predictions, defined as: $Spec=\frac{TN}{\left(TN+FP\right)}$
- Accuracy: represents the overall proportion of correct predictions, given by: $Acc=\frac{\left(TP+TN\right)}{\left(TP+TN+FP+FN\right)}$
- Generalization: measures the model’s ability to correctly identify confirmed YF cases in relation to the total confirmed cases recorded in SISS-Geo, calculated as: $Gen=\frac{TP}{total\_n\_conf}$

We evaluated the Z-Alert index for all clusters with more than one record whose municipalities and event periods match the information in the BMoH database. In this experiment, 492 clusters were assessed using different threshold levels, which define the criticality limit of the clusters (as detailed in section The Z-Alert Index). Table 5 presents the results of performance metrics for different threshold levels (0.8, 0.85, 0.9, 0.95, and 0.99) for the non-dominated solutions obtained in experiments 1, 6, and 11 (see section Analyzing the Pareto Front). These solutions were selected as the most representative based on the prioritization of objectives: solution 1 emphasizes minimizing *f*_2_, solution 6 aims to balance *f*_1_ and *f*_2_, and solution 11 focuses on maximizing *f*_1_. In Table 5, ‘TL’ indicates threshold_level, ‘Alert’ is the number of alerts, ‘NonAL’ represents the number of non-alerts, and ‘PAL’ corresponds to the proportion of alerts. The results in bold show the highest values.

**Table 5:** Performance metrics for the analysis of the Z-Alert Index for experiments 1, 6, and 11 presented in Fig 4.

Sol.	TL	Alert	NonAL	PAL	TP	TN	FP	FN	Prec	Sen	F1	Spec	Acc	Gen
1	0.8	349	143	0.71	188	**124**	161	**19**	**0.54**	0.91	**0.68**	**0.44**	**0.63**	**5.22**
6	0.8	364	128	0.74	193	114	171	14	0.53	0.93	**0.68**	0.40	0.62	5.36
11	0.8	387	105	0.79	**203**	101	**184**	4	0.52	**0.98**	**0.68**	0.35	0.62	**5.64**
1	0.85	341	151	0.69	182	126	159	25	0.53	0.88	0.66	0.44	**0.63**	5.06
6	0.85	355	137	0.72	**189**	119	**166**	18	0.53	**0.91**	**0.67**	0.42	**0.63**	**5.25**
11	0.85	297	195	0.60	159	**147**	138	**48**	**0.54**	0.77	0.63	**0.52**	0.62	4.42
1	0.9	230	262	0.47	131	186	99	**76**	0.57	0.63	0.60	0.65	0.64	3.64
6	0.9	229	263	0.47	**136**	**192**	93	71	**0.59**	**0.66**	**0.62**	**0.67**	**0.67**	**3.78**
11	0.9	232	260	0.47	132	185	**100**	75	0.57	0.64	0.60	0.65	0.64	3.67
1	0.95	132	360	0.27	**78**	231	54	129	**0.59**	**0.38**	**0.46**	0.81	**0.63**	**2.17**
6	0.95	135	357	0.27	77	227	**58**	130	0.57	0.37	0.45	0.80	0.62	2.14
11	0.95	116	376	0.24	66	**235**	50	141	0.57	0.32	0.41	**0.82**	0.61	1.83
1	0.99	24	468	0.05	12	273	**12**	**195**	0.50	0.06	0.10	0.96	0.58	0.33
6	0.99	27	465	0.05	**18**	**276**	9	189	**0.67**	**0.09**	**0.15**	**0.97**	**0.60**	**0.50**
11	0.99	26	466	0.05	17	**276**	9	190	0.65	0.08	**0.15**	**0.97**	**0.60**	0.47

From the results presented in Table 5, we can highlight the following points:

- Proportion of Alerts (PAL) and non-alerts: As the threshold level increases (from 0.8 to 0.99), the proportion of alerts decreases significantly, while the of non-alerts increases. This indicates that, with stricter thresholds, fewer clusters are classified as alerts. For example, at the 0.8 threshold, the PAL ranges from 0.71 to 0.79, while at the 0.99 threshold, PAL drops to 0.05.
- False Negatives (FN): Notice that the worst-case scenario occurs with the false negatives, as this indicates that the system failed to generate an alert for municipalities with confirmed YF cases. As the threshold level increases, false negatives also increase, because with higher thresholds, the model classifies fewer clusters as alerts.
- Precision (Prec): Precision remains nearly constant across all experiments and threshold levels, ranging from 0.52 to 0.65, indicating that, the proportion of correct alerts (with a confirmed YF case) remains relatively stable, with a slight improvement when threshold level increases.
- Sensitivity (Sen): Sensitivity drastically decreases as the threshold level increases. For instance, at the 0.8 threshold, sensitivity ranges from 0.91 to 0.98, while at the 0.99 threshold, it drops from 0.06 to 0.09. This shows that, with stricter thresholds, the model detects fewer alerts with confirmed YF cases, due to the overall reduction in the number of generated alerts.
- F1-Score (F1): The F1-Score, which balances precision and sensitivity, decreases as the threshold level increases, primarily due to a drop in sensitivity. At the 0.8 threshold, F1 is 0.68, while at the 0.99 threshold, it drops to 0.10−0.15. This indicates that, with stricter thresholds, the balance between precision and sensitivity decays.
- Specificity (Spec): Specificity increases with higher threshold levels, ranging from 0.44 (at the 0.8 threshold) to 0.97 (at the 0.99 threshold). This indicates that, with stricter thresholds, the model is better at correctly identifying non-alerts, likely due to the growing number of instances classified as non-alerts. It is important to highlight that specificity depends on false positives and true negatives, which both assume reliable knowledge of where YF did not occur. However, with under-reporting, some true cases go unrecorded. This makes specificity potentially overestimated or misleading.
- Accuracy (Acc): Accuracy is nearly constant across all experiments and threshold levels. This relatively constant values, despite large variations in sensitivity and specificity, may suggest that the model has limited discriminative power, that is, it struggles to effectively separate positive from negative cases. However, a more plausible explanation is that the accuracy metric is being disproportionately influenced by the majority class, probably due to a large number of true negatives, affected by under-reporting and the presence of undiagnosed or unconfirmed cases, an issue also emphasized in [28]. In imbalanced datasets, accuracy can remain misleadingly stable even when the model fails to correctly identify minority-class instances, in this context, true positive cases in disease detection scenarios.
- Generalization (Gen): Generalization decreases as the threshold level increases. This happens because, with stricter thresholds, the model classifies fewer clusters as alerts, resulting in fewer true positives. Here, it is interesting to note the trade-off between generalization and precision. Even though SISS-Geo database contains significantly fewer confirmed YF cases (185 records considered in the optimization process) compared to the BMoH database (3323 records), precision remains stable across all experiments and threshold levels, indicating the model’s robustness to variations in threshold levels.

**Fig 5:**
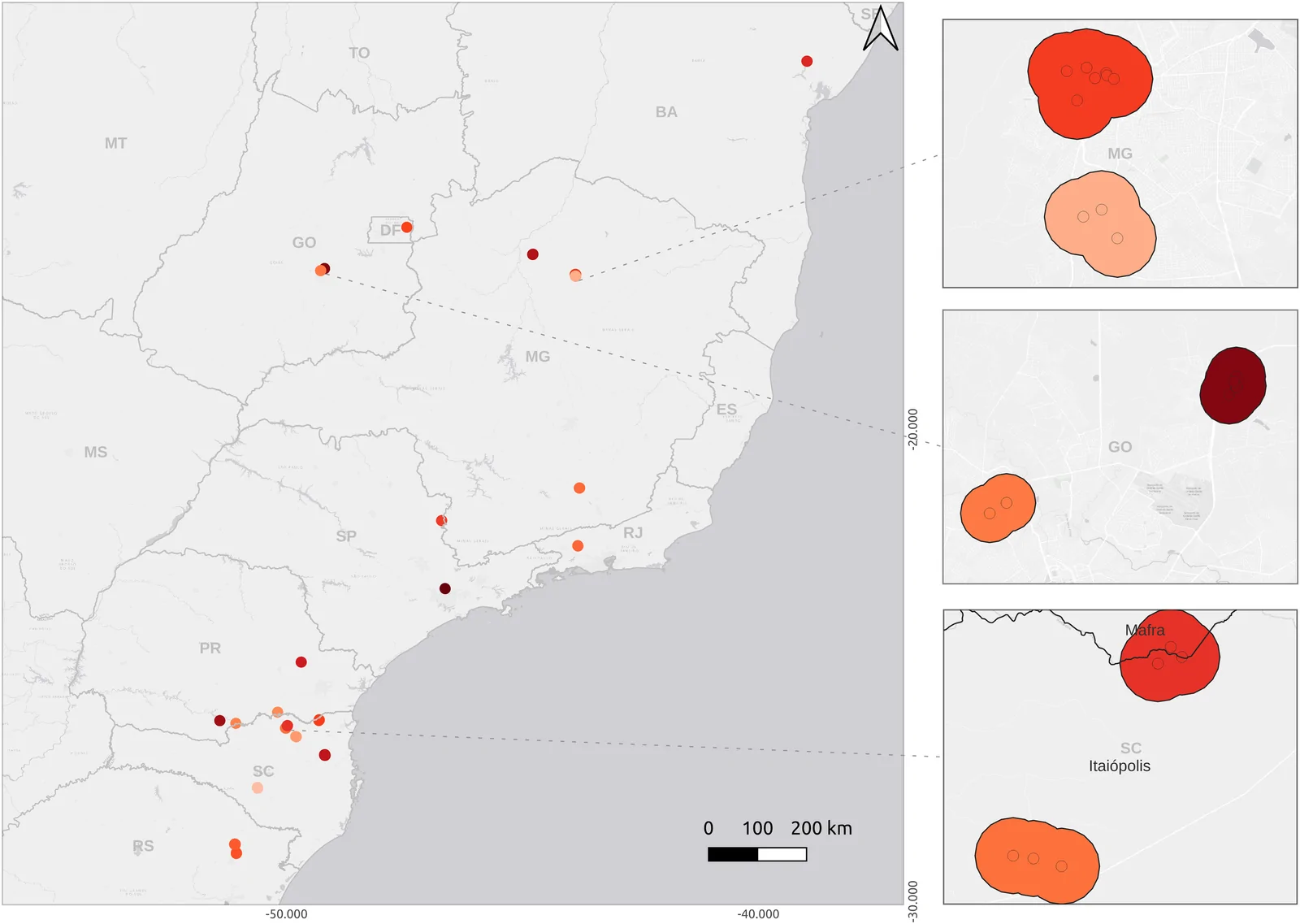
Alert-generating clusters. Result from solution 1 showing 24 alerts at threshold level 0.99. *Map image is the intellectual property of Esri and is used herein under license. Copyright © 2025 Esri and its licensors. All rights reserved. Source: Esri (ArcGIS Online). Tile service: https://services.arcgisonline.com/ArcGIS/rest/services/Canvas/World_Light_Gray_Base/MapServer. Terms of use: https://doc.arcgis.com/en/arcgis-online/reference/static-maps.htm*.

**Fig 6:**
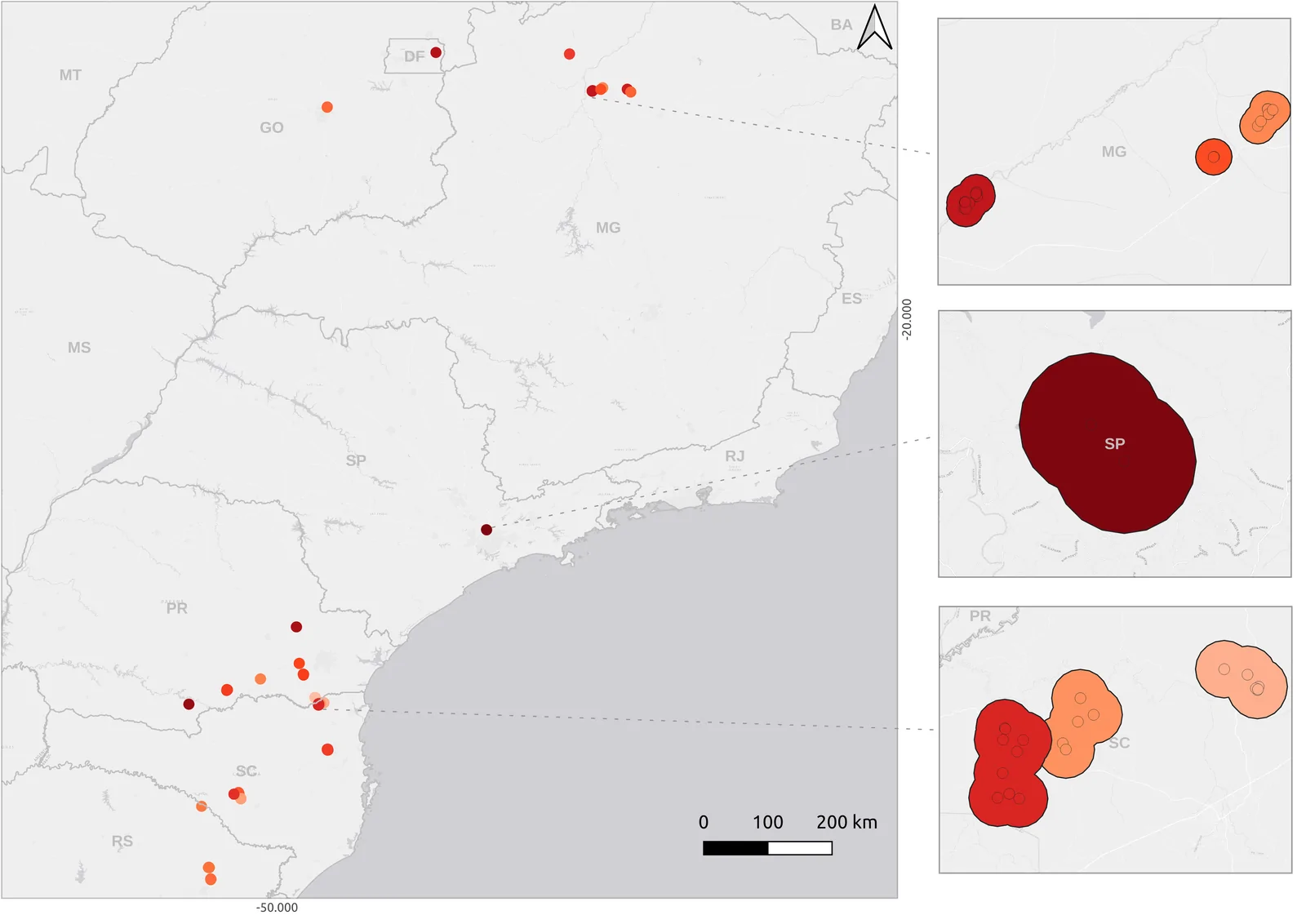
Alert-generating clusters. Result from solution 6 showing 27 alerts at threshold level 0.99. *Map image is the intellectual property of Esri and is used herein under license. Copyright © 2025 Esri and its licensors. All rights reserved. Source: Esri (ArcGIS Online). Tile service: https://services.arcgisonline.com/ArcGIS/rest/services/Canvas/World_Light_Gray_Base/MapServer. Terms of use: https://doc.arcgis.com/en/arcgis-online/reference/static-maps.htm*.

**Fig 7:**
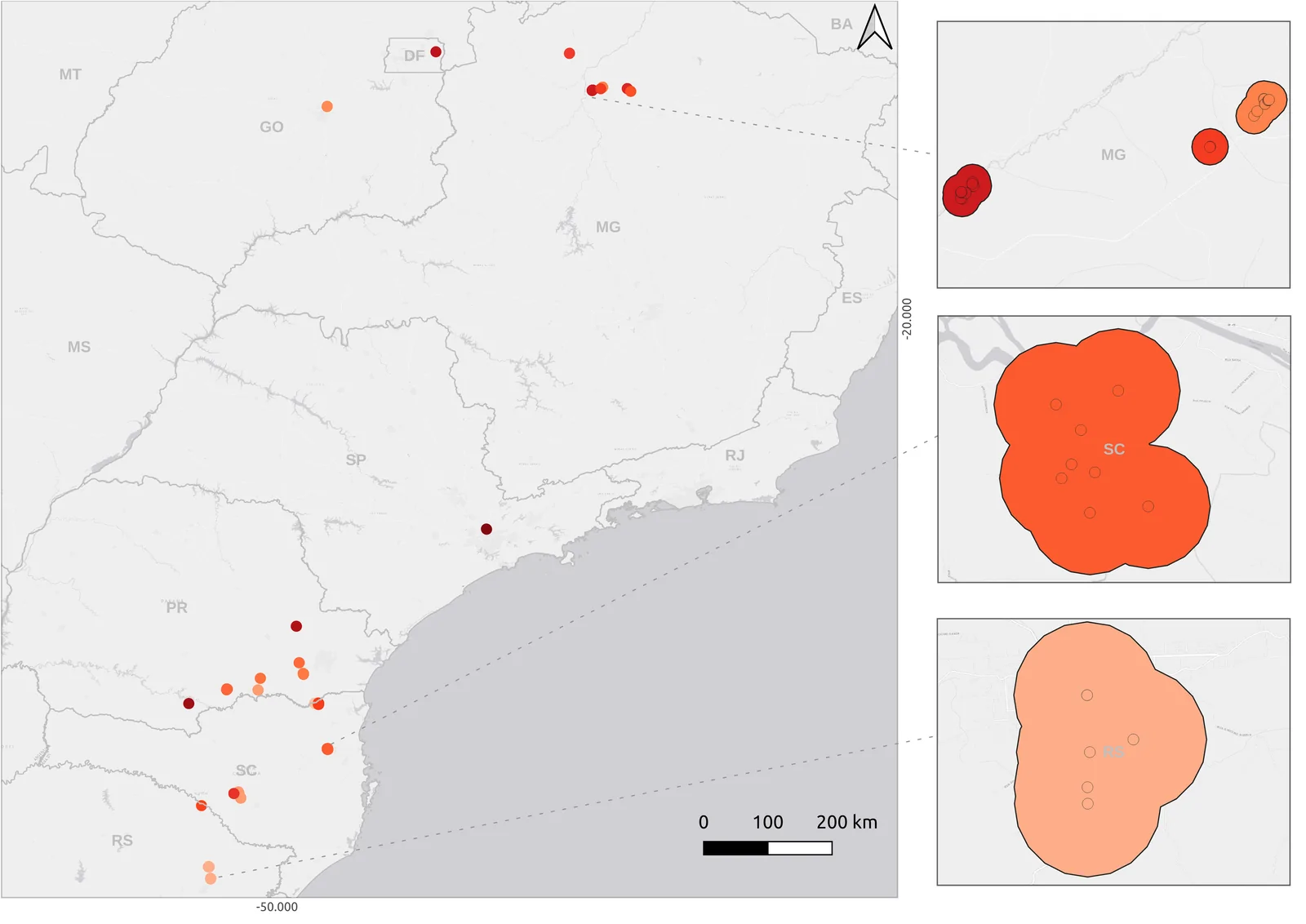
Alert-generating clusters. Result from solution 11 showing 26 alerts at threshold level 0.99. *Map image is the intellectual property of Esri and is used herein under license. Copyright © 2025 Esri and its licensors. All rights reserved. Source: Esri (ArcGIS Online). Tile service: https://services.arcgisonline.com/ArcGIS/rest/services/Canvas/World_Light_Gray_Base/MapServer. Terms of use: https://doc.arcgis.com/en/arcgis-online/reference/static-maps.htm*.

Figs 5, 6,7 illustrate the alert-generating clusters identified in solutions 1, 6 and 11 using a threshold level of 0.99. The color intensity reflects the alert index value, with darker shades indicating higher levels of risk associated with the clusters. The bottom right image in Fig 5 shows a cluster with records located along the border between two municipalities, Mafra and Itaiópolis in Santa Catarina state. In these situations, the alert is generated for both municipalities to ensure comprehensive coverage and response. In all figures, the effect of clustering the points according to the predefined spatial and temporal constraints (see in section Spatiotemporal Distance matrix) is clearly visible, as the events are grouped into coherent regions that reflect both geographic proximity and timing, facilitating more accurate detection and analysis of potential outbreak patterns.

By analyzing the results by solutions, a consistent trend is observed: as the threshold level increases, the number of alerts decreases, leading to higher specificity but lower sensitivity and F1-scores. Some other conclusions can be pointed out:

- Solution 1, which prioritizes uniformity across critical clusters, performance is characterized by the lowest precision (≈0.55 on average) across thresholds, compared with the other solutions. At TL = 0.8, this configuration achieves the heights precision, specificity and accuracy, but these values deteriorate as the threshold increases. This configuration is particularly suited for applications where false alerts carry a high cost, indicated by high specificity across the TL.
- Solution 6 seeks a balanced between the objectives and demonstrates stable and robust performance. At TL = 0.8, it attains good F1-score, with increased performance at TL = 0.9 and 0.99. This configuration provides a good compromise between detection capability and reliability, making it appropriate when both avoiding false negative and capturing true cases are important.
- Solution 11, which aims to emphasize critical clusters, yields the highest sensitivity (0.98) at TL = 0.8 and the highest specificity at TL = 0.95 and TL = 0.99. This model prioritizes avoiding false positives even at the cost of missing true critical clusters. This setting is ideal in scenarios where missing false alerts has a greater cost than issuing true ones.

When comparing results by threshold level, lower TLs (0.8–0.85) result in a higher percentage of alerts (PAL = 0.71–0.79), higher sensitivity (0.91–0.98), and higher F1-scores (0.68), but also with more false positives. Conversely, higher TLs (0.95–0.99) drastically reduce the number of alerts (PAL = 0.27–0.05), improving specificity (up to 0.97 at TL = 0.99) while severely compromising sensitivity (as low as 0.06) and F1-score (as low as 0.10). Higher thresholds are most appropriate when it is critical to avoid false alarms, even if most true cases are missed. Overall, Table 6 presents some recommended choices of threshold level (Rec. TL) based on defined objectives.

**Table 6:** Recommended choices of TL and solution based on defined objectives.

Objective	Rec. TL	Solution	Justification
Maximize detection of real cases	0.8	11	Highest sensitivity (0.98) and F1-score (0.68), minimal false negatives (FN = 4).
Best balance across metrics	0.9	6	More alerts, but all metrics balanced.
Minimize false negative	0.85+	6	On average was the lowest number of false negatives and high performance in all metrics on Rec. TL
Extremely conservative alerts	0.99	Any	Highest specificity (0.96–0.97), very few alerts (PAL = 0.05) and very low false alerts.

### Temporal validation on unseen data

In this section, the Z-Alert Index was evaluated using SISS-Geo records collected between 01 January 2025 and 31 December 2025. The same data collection criteria adopted in the previous experiments were applied:

Animal type: Marmoset and monkey.

Inclusion period: from 01 January 2025–31 December 2025.

Collection date: 26 June 2026.

A total of 2,297 wildlife records were retrieved. These records were subsequently filtered to retain only observations acquired via GPS or with coordinates explicitly provided by the user, with a maximum positional uncertainty of 100 m, resulting in 1,401 records for analysis. After filtering, each record was assigned the corresponding municipality geocode based on its geographic coordinates to support the validation procedure.

The clustering stage employed the same reference configuration adopted in the previous experiments, with spatial and temporal distance thresholds of $L_{D_{S}}=1$ km and $L_{D_{T}}=30$ days, respectively. This process produced 1,037 clusters, of which 848 were single-record clusters (approximately 81% of the total) and were excluded from the subsequent analyses. Therefore, we evaluated the Z-Alert index for all non-unit clusters whose municipalities and event periods match the information in the BMoH database.

The alert index parameters were not recalibrated for this experiment. Instead, the parameter values obtained from the previous study, calibrated using historical data from 05 May 2014–31 December 2024, were used without modification. Consequently, the 2025 dataset served as an independent temporal for evaluating the framework on previously unseen data.

To evaluate whether alerts preceded confirmed YF events, we considered a forward validation window of 30 days following the date on which each alert was generated. For each alert, we verified whether at least one confirmed YF case reported by the BMoH occurred within the corresponding validation window. The BMoH dataset covering the period from 01 January 2025–31 January 2026 was used for this validation, ensuring that alerts generated at the end of 2025 could also be assessed.

In this experiment, 66 clusters were assessed using different threshold levels, which define the criticality limit for classifying a cluster as an alert (see Section The Z-Alert Index). The 66 clusters identified in the independent 2025 dataset were evaluated using the optimized alert index, defined in the previous experiment, and different threshold levels.

Table 7 presents the performance metrics obtained for threshold levels of 0.80, 0.85, 0.90, 0.95, and 0.99, considering the three representative non-dominated solutions selected from the optimization process (Experiments 1, 6, and 11; see Section Analyzing the Pareto Front). These solutions represent different optimization strategies: Solution 1 prioritizes minimizing *f*_2_, Solution 6 provides a balanced trade-off between *f*_1_ and *f*_2_, and Solution 11 prioritizes maximizing *f*_1_. In Table 7, ‘TL’ denotes the threshold level, ‘Alert’ indicates the number of clusters classified as alerts, ‘NonAL’ represents the number of non-alerts, and ‘PAL’ corresponds to the proportion of alerts.

**Table 7:** Performance metrics for the analysis of the Z-Alert Index for experiments 1, 6, and 11 on data from 2025.

Sol	TL	Alert	NonAL	PAL	TP	TN	FP	FN	Prec	Sen	F1	Spec	Acc	Gen
1	0.8	44	22	0.67	23	9	21	13	0.52	0.64	0.57	0.30	0.48	2.88
6	0.8	47	19	0.71	26	9	21	10	0.55	0.72	0.63	0.30	0.53	3.25
11	0.8	48	18	0.73	27	9	21	9	0.56	0.75	0.64	0.30	0.55	3.38
1	0.85	44	22	0.67	23	9	21	13	0.52	0.64	0.57	0.30	0.48	2.88
6	0.85	47	19	0.71	26	9	21	10	0.55	0.72	0.63	0.30	0.53	3.25
11	0.85	48	18	0.73	27	9	21	9	0.56	0.75	0.64	0.30	0.55	3.38
1	0.9	43	23	0.65	23	10	20	13	0.53	0.64	0.58	0.33	0.50	2.88
6	0.9	43	23	0.65	24	11	19	12	0.56	0.67	0.61	0.37	0.53	3.00
11	0.9	37	29	0.56	19	12	18	17	0.51	0.53	0.52	0.40	0.47	2.38
1	0.95	23	43	0.35	16	23	7	20	0.70	0.44	0.54	0.77	0.59	2.00
6	0.95	21	45	0.32	16	25	5	20	0.76	0.44	0.56	0.83	0.62	2.00
11	0.95	21	45	0.32	14	23	7	22	0.67	0.39	0.49	0.77	0.56	1.75
1	0.99	5	61	0.08	3	28	2	33	0.60	0.08	0.15	0.93	0.47	0.38
6	0.99	3	63	0.05	2	29	1	34	0.67	0.06	0.10	0.97	0.47	0.25
11	0.99	3	63	0.05	2	29	1	34	0.67	0.06	0.10	0.97	0.47	0.25

The results obtained using the 2025 dataset exhibit the same overall behavior observed during the previous evaluation. As the threshold level increases, the number of generated alerts decreases, leading to higher precision and specificity at the expense of lower sensitivity. Accuracy remains nearly constant across all threshold levels, whereas the generalization metric gradually decreases. This consistent trade-off indicates that the proposed alert index maintains stable behavior when applied to unseen data.

Similar to the previous experiments, threshold levels around 0.90 provide a balanced compromise between sensitivity and precision, whereas higher thresholds (e.g., 0.95 and 0.99) substantially reduce the number of generated alerts and increase specificity, but also result in a considerable loss of sensitivity.

Among the evaluated solutions, Solution 6 again provided one of the best trade-offs between sensitivity and precision, suggesting that the optimization process identified parameter configurations that generalize well to independent data collected after model development.

**Fig 8:**
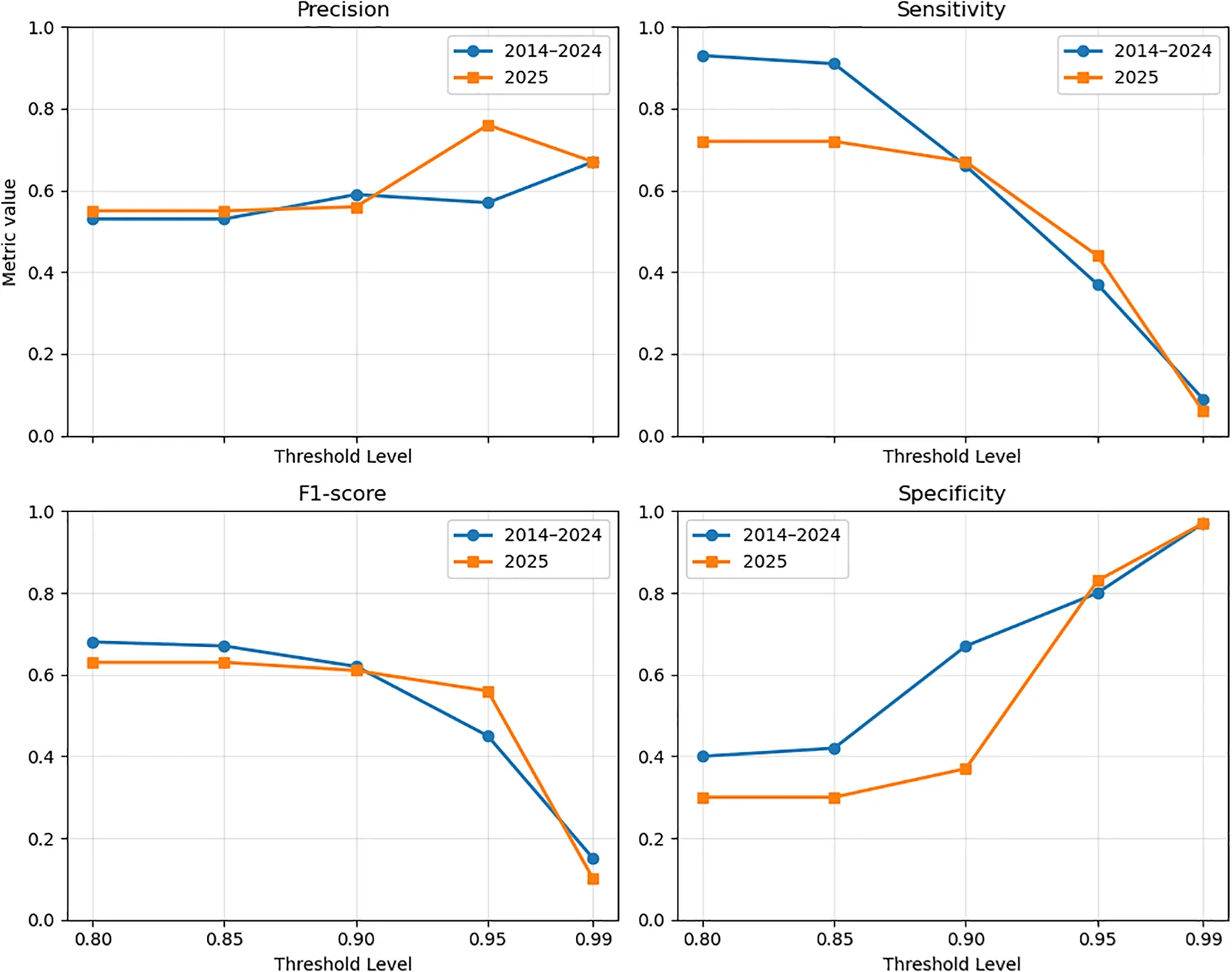
Temporal validation. Comparison of Precision, Sensitivity, F1-score, and Specificity obtained in the previous evaluation (2014–2024) and the temporal validation using unseen SISS-Geo records from 2025 across different threshold levels.

Fig 8 compares the Precision, Sensitivity, F1-score, and Specificity obtained in the previous evaluation (2014–2024) and the temporal validation using data from 2025. The similar trends observed across the different threshold levels indicate that the proposed framework preserves its overall performance when applied to previously unseen data. In both experiments, increasing the threshold level results in higher precision and specificity, and lower sensitivity, while the F1-score exhibits a similar trade-off. These findings provide additional evidence that the optimized alert index generalizes well over time and maintains consistent behavior under independent validation.

These results provide additional evidence that the proposed framework generalizes to future unseen data while preserving the same operational behavior observed during the previous analyses, supporting its use as an early-warning and surveillance-support tool for supporting surveillance teams in prioritizing investigation of relevant events.

### Comparison against the rule-based approach

The fixed rule-based approach represents the current alert generation strategy adopted by SISS-Geo. Under this scheme, alerts are automatically triggered whenever predefined conditions established by domain experts are satisfied, such as reports of dead or sick NHPs or confirmed cases of diseases of interest. For yellow fever surveillance, these rules follow the surveillance guidelines recommended by the BMoH and are intended to ensure that all potentially relevant events are reported to the surveillance network [29]. The current protocol recommends that every report of a dead or sick NHP generates an alert. Consequently, whenever one of these conditions is met, SISS-Geo automatically sends an email notification to the registered public health managers responsible for the corresponding region.

Although SISS-Geo generates alerts since 2019, the systematic monitoring of these notifications began in April 2024. During 2025 alone, these rules generated approximately 14,000 email notifications. Because the rule-based approach relies on binary conditions, all triggered alerts receive the same priority level, regardless of the spatial, temporal, or epidemiological context in which they occur.

In contrast, applying the proposed Z-Alert Index to the independent 2025 dataset resulted in 48 alerts when using a threshold level of 0.80, corresponding to the most exploratory operating configuration evaluated in this study. Rather than notifying every reported event, the proposed framework aggregates spatiotemporal information and multiple cluster attributes to prioritize those events that present higher epidemiological relevance.

This comparison highlights the different operational philosophies of the two approaches. While the rule-based strategy aims to maximize event reporting, it may also generate a very large number of email that compete for the attention of surveillance teams. In contrast, the proposed framework seeks to prioritize alerts according to their level of attention, potentially reducing the operational burden while supporting more efficient allocation of surveillance resources. Although this comparison does not constitute a formal performance benchmark, it illustrates the practical advantage of a prioritization mechanism integrated with the existing SISS-Geo alert system workflow.

It is important to note that the proposed framework is not intended to replace the existing rule-based alerts, but rather to complement them by providing an additional layer for surveillance teams. By integrating multiple cluster attributes and capturing spatiotemporal patterns that cannot be represented by simple rule-based criteria alone, the proposed scheme supports a more informed decision-making process, thereby assisting surveillance teams in allocating investigation efforts and public health resources more efficiently.

## Discussion

Defining the threshold level is essential, as it directly impacts the number of alerts that health managers will receive. In practice, lower threshold values tend to increase sensitivity by generating more alerts, potentially capturing a larger number of true events, while higher thresholds favor specificity by reducing false positives. Therefore, the selection of an appropriate threshold is essential, as it directly influences the balance between detecting relevant events and avoiding excessive or false positive alerts.

From a practical perspective, since this threshold can be adjusted to align with the specific needs and context of each region, it is important to ensure that only the most relevant situations are notified. Setting a higher threshold, resulting in fewer alerts, could be more appropriate in regions with high vaccination coverage and low human mobility, where the risk of disease spread is significantly reduced. If capturing as many alerts as possible is the priority, a lower threshold would be preferred, placing the system in a more exploratory mode aimed at broad surveillance, despite higher false positives may occur. Accordingly, regional surveillance authorities should establish response protocols associated with different alert levels, taking into account local reporting patterns, surveillance capacity, and epidemiological context. Because SISS-Geo records are not uniformly distributed across the national territory, the operational interpretation of alert levels should be adapted to regional characteristics rather than applying a single nationwide criterion.

Based on the Pareto front analyses, the solution that prioritizes the first objective (maximizing *f*_1_) is more effective at identifying real cases, although it may also produce a higher number of false positives. If a balance between detection and precision is required, aiming to reduce extreme variations, the solution seeking a balance between *f*_1_ and *f*_2_ stands out as the most appropriate alternative. On the other hand, if the priority is to minimize false alerts, even at the risk of missing some real cases, the solution that minimizes *f*2 becomes the most suitable choice.

By observing the precision metric, it is possible to conclude that the model has a good ability to generate alerts for clusters with confirmed YF cases, even though this confirmation information is not present in the SISS-Geo database. The optimization process for setting the attribute weights was based solely on data from 38 clusters, which together account for 185 records with confirmed YF cases. This result indicates the model’s strong potential for generalization, successfully identifying critical clusters beyond those used during the optimization process. Notably, the alert index was also able to highlight clusters with high level of attention that did not contain confirmed cases, including some composed solely of live animals. In such cases, the alert system complements the current SISS-Geo rule-based approach, which would not have issued an alert under its fixed criteria.

A key advantage of the alert system proposed is its ability to provide a real-time signal of attention, even in the absence of laboratory confirmation of circulating pathogens among animals. By leveraging spatiotemporal patterns extracted from the data, the index enables early identification of clusters with a high probability of being critical. This proactive approach offers an important decision-support tool for public health management, allowing preventive actions to be taken before zoonotic pathogens are confirmed, thus potentially reducing the time between emergence and response.

Although this study focuses on YF, the proposed methodology is not restricted to this context. The SISS-Geo system also includes records of other zoonotic diseases, such as rabies and avian influenza, suggesting potential for broader applicability. However, extending the framework requires adapting model parameters to the epidemiological and ecological characteristics of each surveillance context. In particular, differences in host species (e.g., primates versus birds), movement patterns, and transmission dynamics may influence both cluster formation and the interpretation of the alert index, reinforcing the need for context-specific calibration and validation.

Despite this potential, the model has important limitations related to the quality and coverage of SISS-Geo data. The effectiveness of the clustering process and the reliability of the Z-Alert index depend on the completeness and accuracy of the records. Moreover, gaps in surveillance, such as delays or failures in reporting confirmed cases and the uneven adoption of SISS-Geo across regions, can further reduce data availability. These issues hinder the model’s ability to detect meaningful patterns and to support the timely prioritization of preventive and investigative actions.

Beyond the limitations associated with data quality, it is also important to consider the scope of the methodological comparisons presented in this study. The proposed framework differs conceptually from traditional spatial analysis methods, such as density-based hotspot detection, kernel density estimation, and other epidemiological cluster detection approaches. These methods are primarily designed to identify geographic areas with high concentrations of events, providing valuable information about the spatial distribution of disease occurrence. In contrast, the objective of the proposed framework is not simply to identify where events are concentrated, but rather to prioritize spatiotemporal clusters according to their epidemiological relevance.

This distinction arises from the different analytical spaces in which the methods operate. Traditional hotspot detection techniques analyze the geographic distribution of individual records, whereas the proposed framework first aggregates records into spatiotemporal clusters and subsequently characterizes each cluster using multiple cluster attributes. A multi-objective optimization procedure is then applied in this multidimensional attribute space to estimate the level of attention associated with each cluster and rank them according to their relative importance. Therefore, these approaches should be regarded as complementary rather than directly interchangeable within surveillance systems.

Nevertheless, we acknowledge that a direct empirical comparison with alternative methods was beyond the scope of the present study. Such comparisons would require defining common evaluation criteria despite the different objectives of these approaches.

## Conclusion

The development of a multi-attribute zoonotic alert index (the Z-Alert Index) represents a significant advancement in epidemiological surveillance integrated within SISS-Geo. By adopting dynamic analyses based on clustering and optimization, the proposed system overcomes the limitations of the current model, enabling proactive identification of critical regions and efficient resource prioritization. Experiments with real-world data demonstrated the index’s ability to detect clusters associated with YF cases with high precision and adaptability to different scenarios. The flexibility to adjust parameters (such as spatial/temporal limits and threshold levels) ensures that the system can be customized according to regional specificities, diseases of interest and public health strategies.

Although the alert index showed a good performance in identifying clusters potentially associated with YF circulation, its applicability to other zoonoses is influenced not only by data availability, but also by confirmed case of diseases, as they play a key role in the optimization process. In terms of data availability, despite SISS-Geo also contains records related to rabies and avian influenza, these are considerably less frequent compared to YF-related reports, which may limit the model’s sensitivity and generalizability in these contexts. While the framework is designed to be adaptable, its transferability to other zoonotic diseases remains to be demonstrated through future studies.

To address these limitations, future work will focus not only on expanding the model’s application to additional zoonoses, such as rabies and avian influenza, by incorporating more data related to these diseases, but also on exploring more generalizable, disease-agnostic alert criteria based on spatiotemporal patterns. In particular, anomaly detection approaches is under investigation [30] to reduce reliance on predefined disease-specific parameters. Additionally, the optimization process will be continuously refined to reduce false negatives and enhance early warning capabilities across a broader range of epidemiological scenarios. Future research will also investigate the influence of the models’ parameters, particularly those related to the clustering stage and the temporal validation window, to better understand their impact on the model’s sensitivity. Moreover, future extensions of this work will examine the incorporation of environmental and climatic variables, such as those presented in [31], which are expected to further strengthen the model’s ability to anticipate zoonotic risks.

It is worth highlighting that regions with efficient monitoring and control strategies tend to attract more investments in technology innovation, and healthcare infrastructure. These benefits reinforce that investing in the identification and prioritization of critical regions is not only a public health concern but also a sound economic strategy. The proposed approach underlines the importance of collaboration among technology, data science, and healthcare management to address complex challenges at the interface of humans, animals, and the environment. In this context, the integration of models designed to identify areas of concern for zoonotic occurrence with monitoring platforms based on citizen-generated information, such as SISS-Geo, represents a key opportunity to strengthen One Health principles by directly connecting wildlife health surveillance with human health contexts.

## Supporting information

S1 Appendix Sensitive analysis of clustering parameters. (PDF)

S1 Fig Pareto fronts generated in the optimization phase for different spatial and temporal clustering parameters. (TIFF)

S2 FigRelative differences in the performance metrics of the evaluated clustering parameterizations compared with the reference configuration ($L_{D_{T}}$=30 days and $L_{D_{S}}$=1 km) across all evaluated threshold levels.Positive values indicate higher performance than the reference, whereas negative values indicate lower performance.(TIFF)
