# Supplementary material for: An index-based system for early alerts of potential zoonotic disease outbreaks
Source: PLoS One. 2026 Aug 25;21(8):e0356739. doi: 10.1371/journal.pone.0356739 (PMC13505946; doi:10.1371/journal.pone.0356739)
Supplement: Supplementary file 1 [file pone.0356739.s001.pdf]

Supporting Information

Jaqueline S. Angelo[1*], Livia Abdalla[1], Douglas A. Augusto[1], Marcia Chame[1], Eduardo Krempser[1],

1 Oswaldo Cruz Foundation (Fiocruz), Rio de Janeiro, RJ, Brazil

* jaqueline.angelo@fiocruz.br

S1 Appendix

In this section, we provide a sensitivity analysis of the clustering parameters L_{D_S} (500 m, 1 km, and 3 km) and L_{D_T} (10 and 30 days), evaluating all six parameter combinations to support the choice of parameter values adopted in the validation experiments.

Table 1 summarizes the clustering results for each parameter configuration. Here, #Clusters denotes the total number of generated clusters, #UClusters the number of unit clusters (single-record clusters), #Confirmed the number of non-unit clusters containing confirmed yellow fever cases, and #VClusters the number of clusters considered during the validation stage.

Table 1. Number of clusters generated for each parameter configuration in the clustering phase.

L_{D_S} (days)	L_{D_T} (km)	#Clusters	#UClusters	#Confirmed	#VClusters
10	0.5	3886	3348	34	369
10	1	3746	2871	37	431
10	3	3286	2638	53	461
30	0.5	3719	3135	34	414
30	1	3508	2841	38	492
30	3	2867	2198	55	490

As expected, reducing the spatial and temporal distance thresholds increases both the total number of clusters and the number of unit clusters, reflecting a more fragmented representation of the data in which fewer records satisfy the clustering criteria. Conversely, increasing these thresholds promotes the aggregation of nearby records, resulting in fewer but larger clusters.

The #Confirmed column is particularly relevant because these clusters are used to calibrate the alert index during the optimization phase. As the spatial and temporal thresholds increase, the number of confirmed non-unit clusters also increases, indicating that confirmed records that would otherwise be split into multiple clusters become grouped into the same epidemiological event.

In contrast, the number of clusters evaluated during validation (#VClusters) varies only moderately across the tested configurations, particularly for L_{D_T}=30 days, suggesting that the choice of clustering parameters primarily affects the composition of the clusters rather than the overall number of events subjected to validation.

Following cluster generation and characterization, the cluster attribute weights are optimized through a multi-objective optimization process. Figure 1 compares the Pareto fronts obtained for the six combinations of spatial and temporal clustering parameters.

We observe that, all parameter configurations produced very similar solutions when minimizing $f2$, with values close to zero. The main differences become apparent as greater importance is assigned to maximizing $f1$. The configuration 10-days/500-m achieved the highest value of $f1$ (approximately 4.5). However, this improvement was accompanied by a substantial increase in $f2$, indicating greater variability in the alert index. In contrast, the configurations 30-days/500-m and 30-days/1-km consistently produced Pareto fronts located below the remaining configurations over most of the optimization range, indicating a more favorable trade-off between maximizing $f1$ and minimizing $f2$. The configurations using L_{D_S}=3 km exhibited a more abrupt deterioration in $f2$ as the optimization increasingly favored $f1$.

Considering that the 30-days/500-m and 30-days/1-km configurations produced the most favorable trade-off between the optimization objectives, and that 30-days/1-km configuration yielded the largest number of confirmed

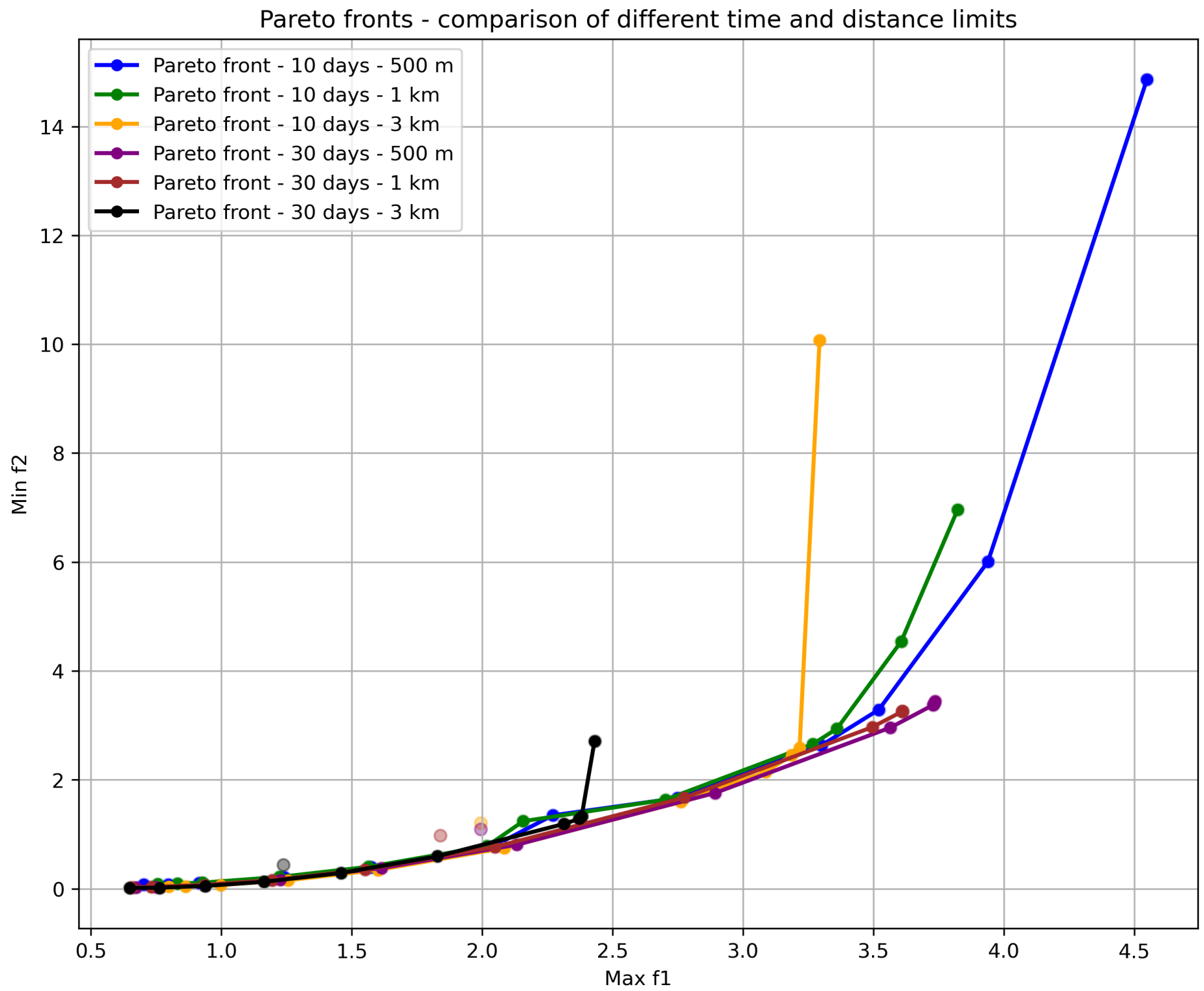

Fig 1. Pareto fronts generated in the optimization phase for different spatial and temporal clustering parameters.

non-unit clusters available for optimization, as shown in Table 1, we selected the L_{D_T}=30 days and L_{D_S}=1 km as the reference parameterization. This configuration provides a balanced compromise between the quality of the Pareto front and the availability of confirmed clusters for constructing and validating the alert index.

Figure 2 summarizes the relative differences in performance with respect to the reference configuration L_{D_T}=30 days and L_{D_S}=1 km. Although some parameterizations produced localized improvements in individual metrics, no alternative parameterization consistently outperformed the reference across all evaluation metrics. Instead, the results reveal the expected trade-offs between Precision, Sensitivity, Specificity, and Generalization.

Configurations using a temporal limit of 10 days generally achieved higher Sensitivity, particularly for intermediate threshold levels, but at the expense of lower Precision and Specificity, indicating an increase in false-positive alerts. Conversely, configurations with larger spatial or temporal limits (30-days/3-km) tended to improve Specificity and Accuracy while reducing Sensitivity and Precision, specially in lower threshold levels. Accuracy exhibited minor variations across the evaluated parameterizations. Similarly, the observed differences in F1-score were not substantial.

Overall, the reference configuration L_{D_T}=30 days and L_{D_S}=1 km provided the most balanced overall performance while also maximizing the number of confirmed non-unit clusters available for optimization. These results support the robustness of the selected clustering parameters given the currently available data. However, as the volume of records and validated cases increases over time, these parameters may be re-estimated through the same sensitivity analysis to ensure that they remain appropriate for the evolving epidemiological scenario.

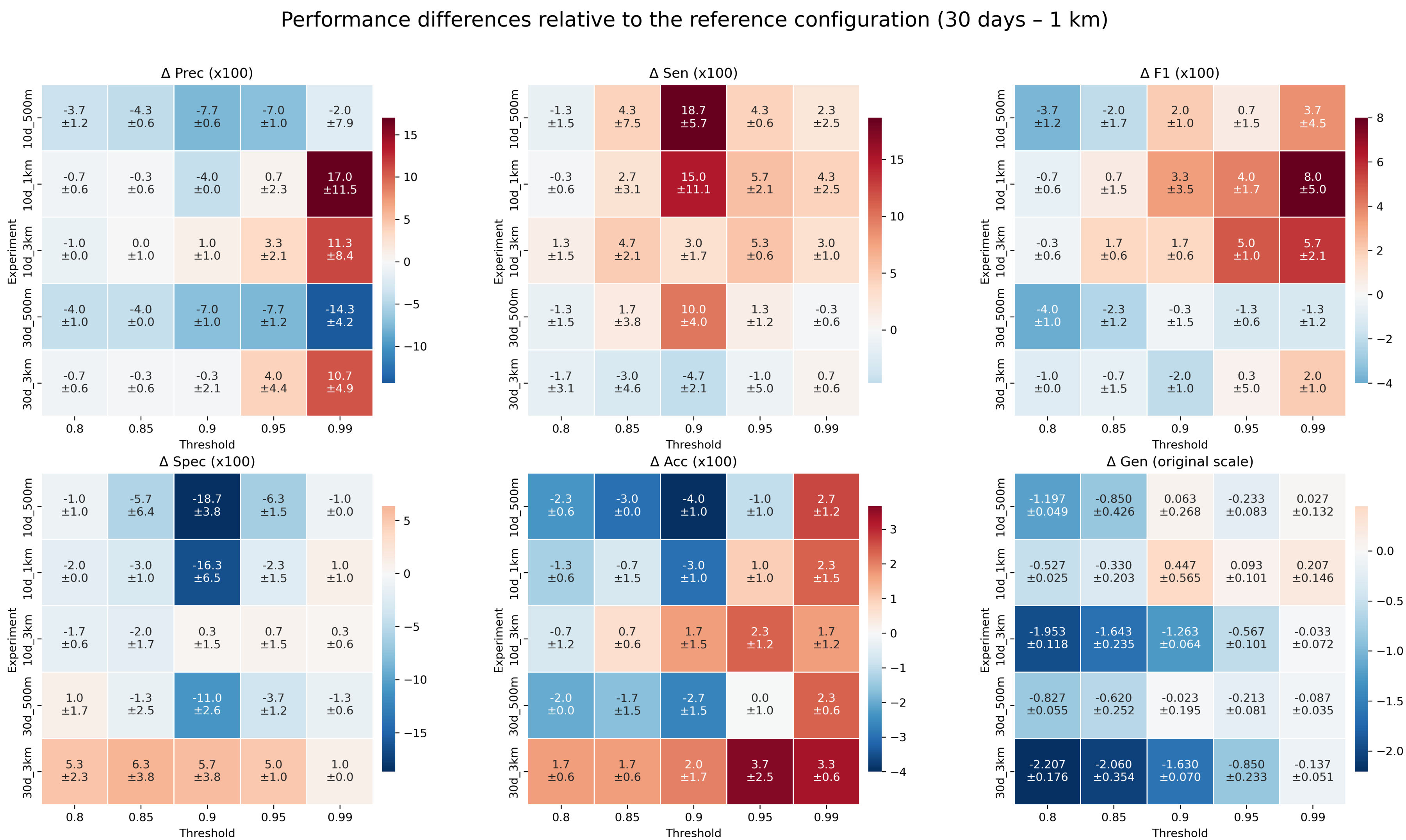

Fig 2. Relative differences in the performance metrics of the evaluated clustering parameterizations compared with the reference configuration (L_{D_T}=30 days and L_{D_S}=1 km) across all evaluated threshold levels. Positive values indicate higher performance than the reference, whereas negative values indicate lower performance.
